# Nucleostemin promotes RAD51 filament assembly on double-stranded DNA to protect stalled replication forks

**DOI:** 10.1093/nar/gkag773

**Published:** 2026-08-10

**Authors:** Chih-Chun Chang, Siang-Sheng Tsai, Chang-Le Chou, Yi-Hsuan Chang, Chia-Tai Hung, Robert Y L Tsai, Hung-Wen Li, Hungjiun Liaw, Peter Chi

**Affiliations:** Institute of Biochemical Sciences, National Taiwan University, Taipei 106, Taiwan; Department of Life Sciences, National Cheng Kung University, Tainan 701, Taiwan; Department of Chemistry, National Taiwan University, Taipei 106, Taiwan; Department of Pharmacy, National Taiwan University, Taipei 106, Taiwan; Institute of Biochemical Sciences, National Taiwan University, Taipei 106, Taiwan; Department of Life Sciences, National Cheng Kung University, Tainan 701, Taiwan; Institute of Biosciences and Technology, Texas A&M University Health Science Center, Houston, TX 77030, United States; Department of Translational Medical Sciences, College of Medicine, Texas A&M University Health Science Center, Houston, TX 77030, United States; Department of Chemistry, National Taiwan University, Taipei 106, Taiwan; Department of Life Sciences, National Cheng Kung University, Tainan 701, Taiwan; Institute of Biochemical Sciences, National Taiwan University, Taipei 106, Taiwan; Institute of Biological Chemistry, Academia Sinica, Taipei 115, Taiwan

## Abstract

Replication stress threatens genome integrity by stalling replication forks, which may lead to double-stranded DNA (dsDNA) breaks. Stalled replication forks can be rescued through the fork reversal mechanism, yet they remain vulnerable to nucleolytic attack. Here, we identify nucleostemin (NS/GNL3) as a critical fork-stabilizing factor. We show that NS rapidly accumulates at hydroxyurea-stalled forks and is indispensable for protecting reversed forks, with NS depletion abolishing RAD51 foci formation and unleashing MRE11-mediated degradation. Through biochemical reconstitution and single-molecule fluorescence resonance energy transfer (FRET), we demonstrate that NS binds DNA directly, engages RAD51, and promotes nucleoprotein filament assembly on the dsDNA segment adjacent to the single-stranded DNA/dsDNA junction. This NS-RAD51 complex synergistically shields nascent strands from MRE11-mediated nucleolytic attack. Our work uncovers a new mechanism of fork protection and positions NS as a key guardian of genome integrity under replication stress.

## Graphical Abstract

**Graphical Abstract:**
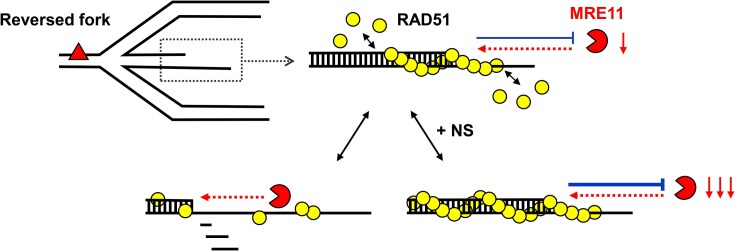

## Introduction

Maintenance of genome integrity requires faithful duplication of the genome by the DNA replication machinery. However, replication forks frequently encounter obstacles such as DNA lesions, replication–transcription collisions, protein–DNA crosslinks, fragile sites, and DNA secondary structures, all of which can impede fork progression and lead to replication stalling [1, 2]. These stresses disrupt normal replication, leading to polymerase-helicase uncoupling and the accumulation of single-stranded DNA (ssDNA). Prolonged ssDNA exposure can lead to double-strand breaks (DSBs), resulting in irreversible fork collapse and genomic instability [3, 4]. To overcome these challenges, several rescue pathways have evolved to restart stalled forks, including repriming synthesis, translesion synthesis (TLS), and fork reversal [5–7]. Repriming synthesis bypasses lesions by initiating PrimPol-mediated DNA synthesis downstream of the damage, while TLS polymerases replicate damaged templates to fill post-replicative ssDNA gaps [8, 9]. However, these pathways are error-prone due to the low fidelity of TLS polymerases, which lack 3′–5′ exonuclease proofreading activity [10, 11]. In contrast, fork reversal is an error-free DNA damage tolerance mechanism that promotes template-switching to facilitate accurate genome duplication [5, 12].

Fork reversal converts a three-way replication fork into a four-way junction, called reversed fork, by annealing newly synthesized strands to form a regression arm. This structure enables the replication process to resume without causing chromosome breakage and allows more time for lesion reconfiguration into double-stranded DNA (dsDNA) for repair [12, 13]. Once replication stress has been resolved, reversed forks can be restarted via RECQ1-mediated branch migration and fork restoration [7, 12, 14, 15]. Controlled resection of the reversed forks is critical for initiating fork restart and triggering homology-directed repair [16, 17]. However, excessive resection by nucleases such as MRE11 can lead to severe fork degradation and genomic instability [16, 18, 19]. Thus, components of the fork protectors, particularly RAD51, are essential for stabilizing and protecting reversed forks [20–24].

RAD51, the recombinase in homologous replication, is essential for regulating reversed fork dynamics [13, 20, 25–29], as highlighted by the finding that RAD51 depletion significantly reduces reversed fork formation. *In vitro* assays and DNA fiber analysis have demonstrated that RAD51 also protects reversed forks [22–24]. It is worth noting that a clinical RAD51 T131P mutant variant, which supports fork reversal but fails to form stable nucleoprotein filaments, is deficient in fork protection, underscoring the critical role of filament stability in safeguarding stalled forks [30, 31].

Nucleostemin (NS/GNL3) was first identified in screens for genes enriched in undifferentiated neural stem cells relative to their differentiated progeny [32]. The NS protein is predominantly localized in the nucleolus under steady-state conditions, but dynamically shuttles between the nucleolus and nucleoplasm via a GTP-regulated mechanism [32–34]. NS protein consists of several key structures, including an N-terminal basic domain, a coiled-coil domain, a highly conserved GTP-binding domain, and a C-terminal acidic domain [32, 35]. Its expression level is generally high in dividing cells but low in nondividing cells [32, 36, 37], which is consistent with its role in self-renewing proliferation and explains the early embryonic lethality observed in NS knockout mice [38–40]. Notably, multiple studies have indicated a role for NS in safeguarding the replicating genome [40–44], despite its paradoxical distribution in the nucleolus. NS depletion markedly elevates γ-H2AX and ATR activation in S-phase cells, reflecting increased fork instability. These damage signals are completely abrogated when cells are shifted into a slower-cycling state by means of serum deprivation [40, 41]. NS also forms nuclear foci following hydroxyurea (HU) treatment, a replication stress inducer, and NS-depleted cells exhibit increased sensitivity to HU [40, 41, 44]. Additionally, NS has been shown to prevent nuclease-mediated resection of nascent DNA during replication stress, likely by suppressing origin firing through the sequestration of ORC2 in the nucleolus [44]. These findings strongly support that NS plays a critical role in protecting replication forks during replication stress.

NS has been implicated in modulating RAD51 [40], which is indispensable for both the formation and protection of reversed forks, under replication stress conditions [15, 20]. NS-deficient cells exhibit markedly diminished RAD51 foci formation following HU treatment, and co-immunoprecipitation assays have confirmed an interaction between NS and RAD51 [40]. However, the mechanism by which NS regulates RAD51 function had been unclear. Here, using complementary cellular, biochemical, and single-molecule approaches, we show that NS accumulates on nascent DNA and protects reversed forks under HU-induced stress. NS binds DNA directly and shields reversed forks from MRE11-mediated degradation. We further demonstrate that NS and RAD51 physically interact and co-localize at stalled forks, where NS recruits RAD51 and synergizes with it to prevent nascent‐strand loss. Our single-molecule fluorescence resonance energy transfer (smFRET) analyses reveal that NS enhances RAD51 filament formation on the dsDNA segment adjacent to the ssDNA/dsDNA junction, thereby promoting filament‐mediated fork protection. These results unveil the role of NS in replication‐stress responses and elucidate its cooperative mechanism with RAD51 in preserving genome stability.

## Materials and methods

### Cells and cell culture

U2OS, T24, and HEK293T cells were obtained from the American Type Culture Collection (VA, USA). U2OS and T24 cells were cultured in McCoy’s 5A medium (Cytiva), and HEK293T cells were cultured in Dulbecco’s modified Eagle medium (Cytiva), both supplemented with 10% fetal bovine serum (FBS, Corning), 1% penicillin, 1% streptomycin, and 1% glutamine, at 37°C and 5% CO_2_.

### RNA interference

Lentiviral particles were generated by co-transfecting HEK293T cells with the plasmids pCMVΔR8.91 and pMD.G, together with pLKO_TRC005 encoding a short hairpin RNA (shRNA) targeting NS, HLTF, SMARCAL1, or BRCA1. All plasmids were obtained from the RNA Technology Platform and Gene Manipulation Core (Academia Sinica, Taipei, Taiwan). The shRNA sequences are listed in Supplementary Table S1. T24 or U2OS cells were infected with shRNA-expressing lentiviruses for 24 h, and subsequently selected with 2 μg/ml puromycin for 1 week. Gene knockdown (KD) efficiency was confirmed by western blotting. Control cells (shLacZ) were generated using the same process, except that lentiviral particles with a nontargeting shRNA were used.

### Western blotting

Cells were lysed in lysis buffer [2% Triton X-100, 1% sodium dodecyl sulphate (SDS), 100 mM NaCl, 10 mM Tris–HCl, pH 8.0, and 1 mM ethylenediaminetetraacetic acid (EDTA)] and sonicated, followed by the addition of Laemmli sample buffer (4% SDS, 125 mM Tris–HCl, pH 6.8, 40% glycerol, 0.01% bromophenol blue, and 10% 2-mercaptoethanol) and boiled at 95°C for 5 min. Protein samples were analyzed by 6%–15% sodium dodecyl sulphate–polyacrylamide gel electrophoresis (SDS–PAGE) and then transferred onto a polyvinylidene difluoride (PVDF) membrane. The membranes were blocked in 5% milk in TBST (20 mM Tris–HCl, pH 7.6, 150 mM NaCl, and 0.1% Tween 20) for 1 h, and then incubated with primary antibodies (Supplementary Table S2) at room temperature for 1–2 h. After incubation with horseradish peroxidase-conjugated secondary antibodies (Supplementary Table S2), membranes were detected using an ECL reagent (PerkinElmer).

### *in situ* protein interaction with nascent DNA replication forks (SIRF) assay

T24 cells were seeded in an eight-well chamber slide (Millipore) at a density of 3 × 10^4^ cells per slide. Cells were pulse-labeled with 10 µM 5-ethynyl-2′-deoxyuridine (EdU, Sigma–Aldrich) for 15 min, and subsequently treated with 2 mM HU (Sigma–Aldrich) for 1–5 h. The nuclear fractions were isolated using an extraction buffer (25 mM HEPES, pH 7.4, 50 mM NaCl, 3 mM MgCl_2_, 1 mM EDTA, 0.3 M sucrose, and 0.5% Triton X-100), and cells were then fixed with 4% paraformaldehyde in phosphate-buffered saline (PBS) for 30 min at room temperature. After click reaction to conjugate EdU with a biotin tag, slides were blocked with PBS containing 10% FBS and 0.1% Triton X-100 for 30 min, followed by incubation with primary antibodies (Supplementary Table S2) against biotin and the protein of interest. Subsequently, samples were incubated with Duolink *In Situ* proximity ligation assay (PLA) probe anti-rabbit PLUS (Sigma–Aldrich, DUO92002) and anti-mouse MINUS (Sigma–Aldrich, DUO92004) antibodies at 37°C for 1 h, followed by the addition of Duolink DNA ligase and polymerase (Sigma–Aldrich). Nuclei were stained with Hoechst 33 258 for 15 min at room temperature and then mounted with ProLong Gold antifade reagent (Invitrogen). To normalize for differences in EdU incorporation among samples, parallel samples were processed for EdU signal quantification. Briefly, cells were pulse-labeled with 10 µM EdU for 10 min and then treated with 2 mM HU for 1 h. Following nuclear extraction and fixing, EdU was conjugated to 1 µM Alexa Fluor 488 azide (Sigma–Aldrich, 760 765) via click chemistry. Samples were washed with PBST (PBS containing 0.05% Tween-20), stained with Hoechst 33 258, and mounted using ProLong Gold antifade reagent. Images were acquired using a Nikon Eclipse 80i microscope equipped with a Plan Fluor 40×/0.75 DIC M/N2 objective and analyzed using ImageJ software (version 1.54s). PLA foci counts were normalized to the median Alexa Fluor 488 intensity measured in the corresponding sample. The resulting values were further normalized to the untreated shLacZ control group and are presented as relative SIRF.

### DNA fiber analysis

T24 or U2OS cells were seeded in 6-cm dishes at a density of 1 × 10^6^ cells per dish. To measure fork progression, cells were pulse-labeled with 25 µM 5-chloro-2′-deoxyuridine (CldU, Sigma–Aldrich) for 30 min, followed by 250 µM 5-iodo-2′-deoxyuridine (IdU, Sigma–Aldrich), and treated with or without 50 µM HU for an additional 30 min. For fork protection assays, cells received identical CldU/IdU pulses, followed by 2 mM HU for 5 h in the presence or absence of 50 µM Mirin (Sigma–Aldrich). Nuclei were isolated by resuspending harvested cells in buffer A (10 mM HEPES, pH 7.9, 10 mM KCl, 1.5 mM MgCl₂, 0.34 M sucrose, 10% glycerol, and 0.1% Triton X-100). Two microliters of nuclear suspension were spotted on glass slides, lysed with 10 µl spreading buffer (0.5% SDS, 50 mM EDTA, and 200 mM Tris–HCl, pH 5.5) for 3–5 min, and allowed to spread by tilting the slide at 15–45°. Fibers were fixed in methanol:acetic acid (3:1) for 10 min, denatured in 2.5 M HCl for 1 h, and blocked with PBS containing 10% FBS and 0.05% Triton X-100. Fixed fibers were incubated with rat anti-5-Bromo-2'-deoxyuridine (BrdU) antibody (Abcam, ab6326, 1:200) for CldU detection for 90 min at room temperature, followed by incubation with goat anti-rat Alexa Fluor 594 secondary antibody (Thermo Fisher Scientific, A-11007, 1:500) for 90 min at room temperature. Fibers were then incubated with mouse anti-BrdU antibody (BD Biosciences, 347 580, 1:200) for IdU detection overnight at 4°C, followed by incubation with goat anti-mouse Alexa Fluor 488 secondary antibody (Thermo Fisher Scientific, A-11001, 1:500) for 90 min at room temperature. Fibers were imaged using a Nikon Eclipse 80i microscope equipped with a Plan Apo 100×/1.40 oil objective. At least 100 fibers per sample were measured with Nikon NIS-Elements (version D4.20.00) and analyzed using GraphPad Prism 10.

### DNA combing analysis

DNA combing analysis was conducted as described previously [45, 46] with some modifications. Briefly, T24 cells were seeded in six-well plates at a density of 3 × 10^5^ cells per well. Cells were sequentially pulse-labeled with 100 µM CldU for 30 min and 100 µM IdU for 30 min, followed by treatment with or without 2 mM HU for 5 h to assess replication fork protection. Approximately 1 × 10^5^ cells were embedded in a 1.2% low-melting-point agarose plug (Bio-Rad). Genomic DNA was isolated by incubating the agarose plugs in ESP buffer (0.41 M EDTA, pH 8.0, 0.91% N-laroylsarcosine sodium salt, and 3.64 mg/ml proteinase K) at 50°C overnight. After washing with TE buffer (10 mM Tris–HCl and 1 mM EDTA, pH 8.0), the plugs were incubated in 0.5 M MES buffer (pH 5.5) for 20 min at 68°C and subsequently digested with β-agarase I (New England Biolabs) at 42°C overnight to ensure complete melting of the plugs. Combing coverslips were coated with octenyltrichlorosilane (Sigma–Aldrich), and genomic DNA fibers were stretched onto silanized coverslips using a DNA combing machine at a constant speed of 300 μm/s. DNA fibers were denatured in denaturing buffer (0.5 M NaOH and 1 M NaCl) for 10 min and then blocked with PBS containing 5% bovine serum albumin (BSA) for 30 min. For immunodetection, DNA fibers were incubated with rat anti-BrdU antibody (Abcam, ab6326, 1:50) for CldU detection for 90 min at room temperature, followed by incubation with goat anti-rat Alexa Fluor 594 secondary antibody (Thermo Fisher Scientific, A-11007, 1:100) for 90 min at room temperature. Fibers were then incubated with mouse anti-BrdU antibody (BD Biosciences, 347 580, 1:25) for IdU detection overnight at 4°C, followed by incubation with goat anti-mouse Alexa Fluor 488 secondary antibody (Thermo Fisher Scientific, A-11001, 1:100) for 90 min at room temperature. DNA fibers were imaged using a Nikon Eclipse 80i microscope equipped with a Plan Fluor 40×/0.75 DIC M/N2 objective. Images were analyzed using NIS-Elements D software (version 4.20.00).

### Immunofluorescence

Cells were seeded in eight-well chamber slides (Millipore) at a density of 1 × 10^5^ cells per slide. Following pulse labeling with 10 µM EdU, cells were treated with 2 mM HU for either 5 h or 24 h. Nuclear fractions were isolated using the extraction buffer, and subsequently fixed with 4% paraformaldehyde for 10 min at room temperature. EdU-labeled DNA was detected by conjugation with 20 µM Alexa Fluor 488 azide using a click chemistry reaction. Samples were blocked with blocking buffer (PBS containing 10% FBS and 0.05% Triton X-100) for 30 min at room temperature and then incubated with anti-RAD51 antibodies overnight at 4°C. After washing three times with PBS, cells were incubated with Alexa 594-conjugated secondary antibodies for 1 h at room temperature. Nuclei were stained with Hoechst 33 258 for 15 min at room temperature following PBS washing. Finally, slides were mounted using ProLong Gold antifade reagent (Invitrogen). Images were acquired using a confocal laser scanning microscope (Olympus FV3000) and analyzed using ImageJ software.

### Cloning, expression, and purification of proteins

#### Human NS recombinant protein

Human NS cDNA was cloned into the pMAL-c5X vector (New England Biolabs) via NotI/SalI with an N-terminal maltose‐binding protein (MBP) tag and a C-terminal His₆ tag. The resulting plasmid was transformed into *Escherichia coli* Rosetta (DE3) pLysS, and protein expression was induced with 0.2 mM isopropylthio-β-galactoside (IPTG) at 16°C for 16 h. All subsequent steps were carried out at 4°C. Cell pellets were resuspended in buffer B (25 mM Tris–HCl, pH 7.5, 10% glycerol, 300 mM KCl, 0.01% Igepal-CA-630, 0.5 mM EDTA, and 1 mM β-mercaptoethanol) containing protease inhibitors—2 mM phenylmethylsulfonyl fluoride (PMSF), 2 mM benzamidine, and 1 µg/ml each of aprotinin, chymostatin, leupeptin, and pepstatin A—and lysed by sonication. After clarification at 40 000 × *g* for 1 h, the supernatant was treated with 0.25% polyethyleneimine (Sigma–Aldrich) for 30 min to precipitate nucleic acids, then transferred to 242 mg/ml ammonium sulfate (Bio Basic) to selectively precipitate NS. The pellet was resuspended in buffer B and incubated with Ni-NTA agarose (QIAGEN) overnight. The resin was then washed with buffer B containing 1 M KCl, followed by buffer B containing 500 mM KCl. MBP-NS-His₆ was eluted with buffer B supplemented with 200 mM imidazole. Finally, the eluate was further fractionated by size-exclusion chromatography on a Superdex 200 Increase 10/300 GL column (GE Healthcare) in buffer B to remove soluble aggregates. The peak fractions were concentrated, aliquoted, and stored at −80°C.

#### Human RAD51 recombinant protein and its mutant variants

Recombinant human RAD51 (wild-type) and the K133A and Y232W mutant variants were expressed in a RecA-deficient *E. coli* BLR strain as previously described [47], and the purification protocol was modified from the same study. In brief, clarified lysates underwent 35% ammonium sulfate precipitation, followed by sequential chromatography on Sepharose Q (GE Healthcare), hydroxyapatite (Bio-Rad), Source Q (GE Healthcare), and Mono Q (GE Healthcare) columns. The T131P variant was purified using our established protocol with Talon affinity (Takara), Source Q, Heparin (GE Healthcare), and Mono Q chromatography [25].

#### Human MRE11 recombinant protein

Human MRE11 was expressed in human Expi293F cells (Thermo Fisher) and purified as described previously [48]. In brief, cell lysates were subjected to sequential affinity chromatography on Ni-NTA and anti-Flag M2 agarose resins (Sigma–Aldrich), followed by anion-exchange chromatography on a Mono Q column. Peak fractions were pooled, concentrated, aliquoted, and stored at −80°C.

### DNA substrates

The DNA oligonucleotides used in this study (sequences listed in Supplementary Table S3) were annealed to generate the indicated substrates. To prepare substrates for the electrophoretic mobility shift assays (EMSA) and MRE11 protection assay, fluorescently labeled oligonucleotides (Genomics) were annealed with complementary nonmodified or 5′/3′-phosphorothioate-modified oligonucleotides (IDT) at a molar ratio of 1:1.2 in annealing buffer (50 mM Tris–HCl, pH 7.5, 10 mM MgCl₂, 100 mM NaCl, and 1 mM Dithiothreitol). The terminal phosphorothioate modifications protect the substrates from nonspecific nuclease digestion. Duplex DNA was generated by annealing oligo 1 with oligo 2; the 5′- and 3′-overhang substrates were formed by pairing oligo 3 with oligo 4 or oligo 5, respectively; and the reversed fork-mimicking DNA was assembled by mixing oligos 6–9. Annealing reactions were performed by heating to 80°C for 3 min, incubating at 65°C for 30 min, and then cooling gradually to room temperature. Substrates were purified from Tris–borate–EDTA polyacrylamide gel electrophoresis (TBE–PAGE) gels by electro-elution, concentrated, and buffer-exchanged into TE buffer (10 mM Tris–HCl, pH 8.0, and 0.5 mM EDTA) at 4°C using Amicon ultra-4 centrifugal filters (Millipore, NMWL 10 kDa). smFRET substrates were prepared similarly with oligos 10–14 (dye pairs at or away from the ssDNA/dsDNA junction) in Elution Buffer NE (MACHEREY-NAGEL). Oligos 10–14 were purchased from IDT, and sulfo-Cy3 NHS ester (Lumipore) was labeled onto oligos 12 and 14 via amine-NHS ester conjugation.

### Electrophoretic mobility shift assays

The indicated amount of NS protein was incubated with ssDNA (oligo 2), dsDNA, 5′-overhang, 3′-overhang, or reversed fork-like substrate (58 nM molecules) in 10 μl of buffer C (35 mM Tris–HCl, pH 7.5, 1 mM DTT, 50 mM KCl, 0.1 μg/μl BSA, 2.5 mM MgCl_2_, and 1 mM ATP) at 37°C for 10 min. The reaction mixture was resolved on a 0.6% TBE–agarose gel with 1 × Tris–borate–EDTA (TBE) buffer at 80 V for 50 mins. Gels were imaged using an Amersham^TM^ Typhoon^TM^ Biomolecular Imager (Cytiva) with a Cy3 570BP20 560–580 nm filter.

### MRE11 protection assay

A fluorescently labeled 5′-overhang DNA substrate (58 nM) was sequentially incubated with 300 nM RAD51 or *E. coli* RecA (New England Biolabs) for 10 min, NS at the indicated concentration for an additional 10 min, and then MRE11 (400 nM) for 40 min in 10 µl buffer C supplemented with 1 mM MnCl₂ at 37°C. Reactions were terminated by adding 2.5 µl termination buffer (0.4% SDS, 0.24 M EDTA, and 3 mg/ml proteinase K) and incubating at 37°C for 15 min. An equal volume of 2 × denaturing dye (95% formamide, 0.1% Orange G, 10 mM Tris–HCl, pH 7.5, 1 mM EDTA, and 12% Ficoll PM400) was added, and the samples were heated at 95°C for 10 min. Products were resolved on a 27% denaturing TBE–urea gel (7 M urea) with 1 × TBE buffer at 300 V at 55°C for 50 min, and then imaged using an Amersham^TM^ Typhoon^TM^ Biomolecular Imager with a Cy3 570BP20 560–580 nm filter.

For reversed fork-mimicking substrates (29 nM), RAD51 and NS incubations were performed as described above, and the MRE11 digestion was extended to 80 min in buffer C containing 2.5 mM MnCl₂. Termination, denaturation, electrophoresis, and imaging were conducted as detailed above.

### Affinity pull-down assay

Affinity pull-down assays were performed by incubating NS (0.7 µg) with RAD51 (0.7 µg) in 10 µl buffer D (25 mM Tris–HCl, pH 7.5, 10% glycerol, 50 mM KCl, 2.5 mM MgCl₂, 1 mM ATP, and 20 mM imidazole) at 37°C for 20 min. Samples were then mixed with 5 µl His-tag Dynabeads (Invitrogen) and incubated for an additional 20 min at 37°C. Beads were captured on a magnetic separator, and the supernatant (unbound fraction) was collected. Bound proteins were eluted in 10 µl 2× SDS loading buffer by heating at 95°C for 5 min. Both unbound and eluted fractions were resolved on 10% SDS–PAGE and visualized by Coomassie Blue staining. Gels were imaged using a Gel Doc XR + system with Image Lab software 6.0 (Bio-Rad).

### Single-molecule fluorescence resonance energy transfer

smFRET experiments were performed as described previously [49, 50]. In brief, slides were passivated with polyethylene glycol (PEG) and PEG-biotin to reduce nonspecific protein binding on slides and to allow DNA attachment. Each slide chamber was incubated with 0.01 mg/ml streptavidin for 2 min, followed by a wash with buffer E (20 mM Tris–HCl, pH 8, and 50 mM NaCl). Next, 5–10 pM of biotin-labeled DNA was flowed into the chamber for 2 min, and the free DNA was then washed out with image buffer (35 mM Tris–HCl, pH 8, 50 mM KCl, 2.5 mM MgCl_2_, 1 mg/ml BSA, 2 mM Trolox, 4 mg/ml glucose, 60 U/ml glucose oxidase, 2 mM cyclooctatetraene (COT), and 1 mM DTT). RAD51 (50 nM) and NS (8 nM) were mixed in 20 μl image buffer, loaded into the chamber, and fluorescent images were acquired.

A micro-mirror-based total internal reflection fluorescence microscope (RM21, Mad City Labs) with a 532-nm laser (0532L-21B, MatchBox) and a 635-nm laser (Radius 635-25, Coherent) was used for imaging acquisition [51]. Fluorescence was split by the band-pass filters (ZET532/640m, Chroma) and collected by a dual-view system (Optosplit II bypass, Cairn Research; FF640-FDi01, Semrock) with an EMCCD camera (iXon Ultra 888, Andor). Automatic drift correction was done using a quadruple photodiode (PSM 2–10Q, On-Trak Photonics) and a piezo-electric stage (Mad City Labs).

The fluorescence images were acquired using a custom-written Glimpse program (modified from Jeff Gelles’ lab, https://github.com/gelles-brandeis/Glimpse). For real-time recording, a 532-nm laser (1.3 mW) was used to record 2800 frames immediately after loading the protein solution into the chamber, followed by a 635-nm laser (0.9 mW) to record 200 frames with an exposure time of 0.1 s. For snapshot recording, a 532-nm laser (1.3 mW) was used to acquire 100 frames with an exposure time of 0.5 s, 5 min after the protein solution had been loaded into the chamber.

Image analysis was performed using custom-written Python scripts. Colocalized spots from the red and green channels were identified and confirmed for single-molecule analysis. The FRET values were averaged by a moving window of 5 frames for snapshots or 25 frames for real-time analysis. The DNA-only state was bounded as [µ − 2σ, µ + 2σ], where µ and σ were acquired from Gaussian distribution fitting of the DNA-only group. Data from each condition were collected from three independent experiments (*N* = 3), each of which contained more than 60 qualified molecules (*n* > 60).

### Statistics

All statistical analyses of cell-based and biochemical data were performed using GraphPad Prism 10 (GraphPad software). The Mann–Whitney test was used to assess significance in the SIRF, DNA fiber, immunofluorescence, and DNA combing assays. Independent biological replicate experiments for SIRF and DNA fiber assays shown in the main figure are provided in Supplementary Fig. S10. DNA-binding and protection levels in the EMSA and MRE11 assays were quantified in ImageJ and normalized to the corresponding controls. Data normality was assessed by the Shapiro–Wilk test. The sample variance was confirmed to be similar between groups by the Brown–Forsythe test. One-way analysis of variance (ANOVA) with Tukey’s post hoc test was used for comparisons of multiple groups to determine statistical significance. The smFRET statistics were performed by Python scripts. Bootstrapping 5000 times for each group was performed. Student’s *t*-test was performed using the statannotations package. A *P*-value <0.05 was considered statistically significant.

## Results

### NS is recruited to stalled forks in response to replication stress

**Figure 1.**
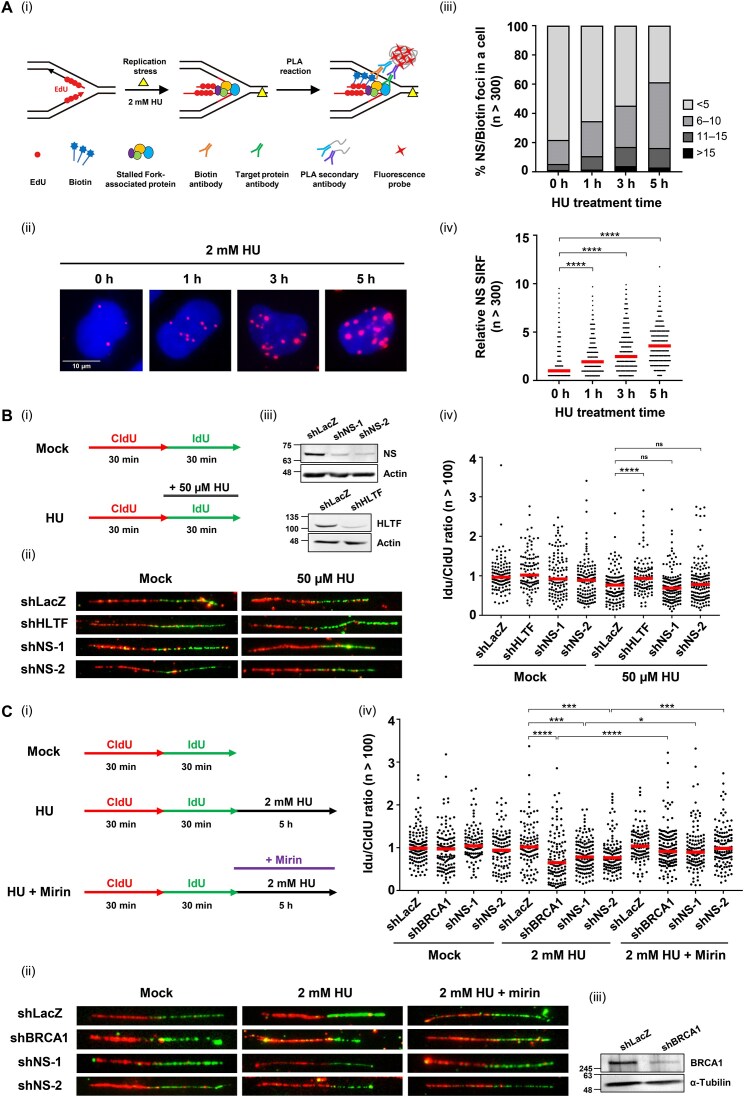
NS is enriched at HU-induced stalled forks to protect nascent DNA from MRE11-mediated degradation. (**A**) Single-cell SIRF-based quantification of NS at stalled forks. (i) Schematic of the SIRF experimental design. EdU-labeled nascent DNA was biotinylated via click chemistry, probed with anti-biotin and anti-NS antibodies, and then visualized by proximity ligation. (ii) Representative images of NS-biotin PLA foci (red) and Hoechst-stained nuclei (blue) in cells treated with or without 2 mM HU for 1–5 h. (iii) The number of PLA foci per nucleus was binned into four categories (<5, 6–10, 11–15, >15 foci), and the percentage of cells in each category is plotted. (iv) Quantification of PLA foci per nucleus was analyzed from >300 cells for each condition. Relative SIRF was calculated by normalizing PLA foci per cell to the median Alexa Fluor 488 signal of the corresponding sample (Supplementary Fig. S1D and E) and then to untreated cells. All *P*-values between the four treatment groups are <0.0001. (**B**) Replication fork progression under 50 µM HU challenge. (i) Schematic of the DNA-fiber experimental design. Wild-type, HLTF-knockdown (KD), or NS-KD cells were pulse-labeled with CldU and then with IdU in the presence or absence of 50 µM HU. (ii) Representative images of CldU (red) and IdU (green) tracts. (iii) Western blots of HLTF and NS in control-KD (shLacZ), NS-KD (shNS-1, shNS-2), and HLTF-KD cells. (iv) Quantification of IdU:CldU tract-length ratios from > 100 fibers per condition. *P*-value between shLacZ and shHLTF is <0.0001; for the shNS-1 group it is ns (not significant): *P* = 0.6511; and for the shNS-2 group it is also ns: *P* = 0.1988. (**C**) Fork stability during prolonged HU exposure. (i) Schematic of the DNA-fiber experimental design. Cells in the wild-type, BRCA1-KD, or NS-KD backgrounds were labeled with CldU and IdU, and then treated with 2 mM HU ± 50 µM Mirin. (ii) Representative fiber images. (iii) Western blot of BRCA1 in control-KD (shLacZ) and BRCA1-KD cells. (iv) IdU:CldU ratios quantified as in panel (A) from >100 fibers. Under HU treatment, the *P*-value between the shLacZ group and the shBRCA1 group is <0.0001; for the shNS-1 group it is 0.0006; and for the shNS-2 group it is 0.0004. The *P*-value between HU treatment and HU and Mirin co-treatment of shBRCA1 groups is <0.0001; for the shNS-1 groups it is 0.0202; and for the shNS-2 groups it is 0.0003. All statistical significance values were determined by Mann–Whitney tests. The red line indicates the median.

To establish the direct role of NS in repairing replication-associated DNA lesions, we employed an *in situ* protein interaction with nascent DNA replication forks assay (SIRF) to assess its recruitment to stalled forks under replication stress at single-cell resolution. In the SIRF assay (Fig. 1A, panel i), nascent DNA was pulse-labeled with EdU and then stalled by HU treatment. EdU was subsequently conjugated to biotin via click chemistry. Cells were then probed with antibodies against biotin and NS, and a PLA was used to visualize NS protein in proximity to the EdU-labeled stalled forks. To validate the system, we employed a combination of mouse and rabbit anti-biotin antibodies (biotin M/biotin R) and an anti-proliferating cell nuclear antigen (PCNA) antibody (PCNA/biotin) as positive controls, both of which produced abundant PLA foci (Supplementary Fig. S1A–C). In contrast, no PLA signals were observed when only an anti-NS or anti-biotin antibody was applied or when antibodies against the unrelated microtubule-associated protein EB1 (EB1/biotin) were used, confirming the resolution and specificity of our analysis (Supplementary Fig. S1A–C). The SIRF analysis showed that, under unperturbed conditions, ∼20% of cells exhibited more than five NS-biotin PLA foci at replication forks (Fig. 1A, panels ii and iii). Upon HU-induced fork stalling, NS recruitment increased in a time-dependent manner (Fig. 1A, panel ii–iv). After 3 h of HU treatment, over 40% of cells displayed more than five NS-biotin PLA foci and, by 5 h, NS localization had risen threefold, with ∼60% of cells harboring more than five foci (Fig. 1A, panel iii). Notably, NS depletion virtually abolished NS-biotin PLA foci regardless of HU treatment, validating the specificity of NS detection by the SIRF assay (Supplementary Fig. S1F–H). Furthermore, to determine whether fork reversal contributes to NS recruitment, we depleted SMARCAL1, a key mediator of fork remodeling [52], using two independent shRNAs. Our results show that SMARCAL1 depletion significantly reduced NS recruitment to stalled forks following HU treatment (Supplementary Fig. S2). Together, our SIRF analysis demonstrates that NS associates with reversed forks and undergoes significant recruitment to the nascent DNA of stalled forks in response to replication stress, indicating its direct role in the cellular response to fork stalling.

### NS depletion results in nascent DNA degradation during replication stress

Remodeling stalled replication forks into reversed forks is known to prevent fork collapse and facilitate restart [15, 20]. Next, we tested if NS contributes to fork reversal by means of a DNA fiber analysis. To do so, T24 cells were sequentially pulse-labeled with CldU for 30 min, followed by IdU for 30 min in the presence or absence of HU (see Fig. 1B, panel i, for a schematic diagram). Stretched DNA fibers were then immunostained for CldU and IdU, and the IdU:CldU tract-length ratio was calculated as a measure of replication speed (Fig. 1B, panel ii). We analyzed two independent NS KD lines, as well as HLTF-depleted cells (used as a positive control for fork reversal). KD efficiencies were confirmed by western blotting (Fig. 1B, panel iii). Under normal cell growth conditions, all samples displayed an IdU:CldU ratio close to 1, indicative of normal fork progression (Fig. 1B, panel iv). Upon HU treatment, the shLacZ control showed a reduced IdU:CldU ratio (∼0.8), consistent with fork reversal reducing fork speed, whereas depletion of the fork remodeler HLTF restored the ratio to close to 1 [53, 54]. Strikingly, NS‐depleted cells exhibited the same HU‐induced decrease in IdU:CldU ratio as control cells, demonstrating that NS is not required for reversed fork formation under the HU treatment condition (Fig. 1B, panel iv).

Prolonged exposure of reversed replication forks renders them vulnerable to nuclease‐mediated degradation by MRE11 [18, 23, 24]. To assess if NS protects these structures, we performed DNA fiber assays in which cells were sequentially pulse-labeled with CldU and IdU for 30 min each, then treated with HU for 5 h to induce fork reversal (see Fig. 1C, panel i, for the schematic diagram). Measurement of IdU:CldU tract‐length ratios (Fig. 1C, panel ii) revealed that, under the normal growth condition, fibers maintained a ratio close to 1 (Fig. 1C, panel iv). Depletion of BRCA1 (a known fork protector) reduced this ratio to ∼0.6 upon HU treatment (Fig. 1C, panels ii–iv), confirming its role in preventing nascent‐strand degradation [22, 55]. Importantly, NS KD similarly lowered the IdU:CldU ratio to ∼0.8, indicative of enhanced degradation of reversed forks in the absence of NS. Comparable results were also obtained from U2OS cells (Supplementary Fig. S3A–C). Furthermore, to confirm that the observed fork protection defect was specifically attributable to NS loss, we performed complementation experiments by re-expressing FLAG alone or FLAG-tagged NS in NS-depleted cells (Supplementary Fig. S3D and E). Notably, whereas NS depletion reduced the IdU:CldU ratio following HU treatment, re-expression of FLAG-NS, but not FLAG alone, restored the ratio and rescued the nascent-strand degradation phenotype (Supplementary Fig. S3E). Moreover, consistent with the observed fork protection defect, NS-depleted cells exhibited increased sensitivity to HU treatment and reduced survival compared with control cells (Supplementary Fig. S3F).

Since unprotected regressed arms provide entry points for MRE11, we tested if Mirin-mediated MRE11 inhibition could rescue fork degradation. Co‐treatment with Mirin restored the IdU:CldU ratio close to 1 in both BRCA1‐ and NS‐depleted cells under HU treatment, confirming that the observed DNA loss is MRE11-dependent (Fig. 1C, panels ii–iv). These results are in line with a recent report showing that NS limits resection at stalled forks [44]. Collectively, these findings demonstrate that NS functions as a crucial protector of reversed forks, safeguarding nascent DNA from MRE11‐mediated degradation instead of facilitating the formation of reversed forks.

### Expression and purification of recombinant NS protein

**Figure 2.**
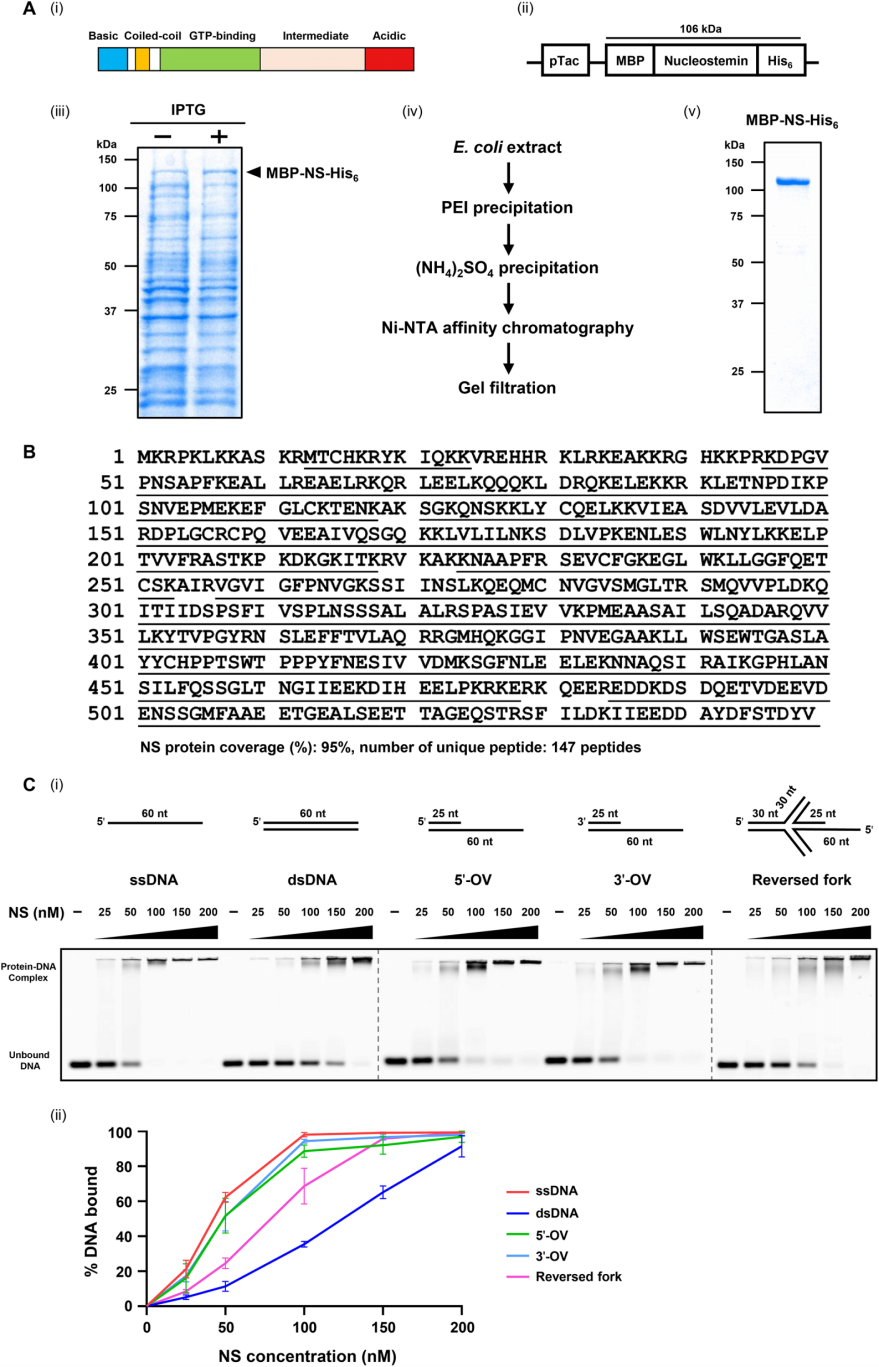
Purified recombinant NS protein possesses DNA-binding activity. (**A**) Expression and purification of MBP-NS-His_6_ protein. (i) Schematic diagram of NS protein domain architecture. (ii) Schematic diagram of the MBP-NS-His₆ construct. (iii) Coomassie-stained SDS–PAGE of *E. coli* lysates before (–) and after (+) IPTG induction. (iv) Purification procedure. (v) Coomassie-stained SDS–PAGE of the final purified NS protein. (**B**) Liquid chromatography–tandem mass spectrometry (LC-MS/MS) confirmation of NS identity. Underlined peptides indicate 95% sequence coverage. (**C**) NS DNA-binding activity. (i) Electrophoretic mobility shift assays using 58 nM Cy3-labeled substrates (ssDNA, dsDNA, 5′-overhang, 3′-overhang, and a reversed fork) incubated with increasing concentrations of NS. (ii) Quantification of bound DNA from panel (i), based on three independent experiments [mean ± standard deviation (SD)].

To enable *in vitro* characterization of NS-mediated fork‐protection activity, we generated a human NS construct bearing an N‐terminal MBP tag and a C‐terminal hexahistidine (His₆) tag to enhance solubility and allow affinity purification (Fig. 2A, panel i and ii). Recombinant MBP-NS-His₆ was expressed in *E. coli* (Fig. 2A, panel iii), and the clarified lysate was treated with polyethyleneimine (PEI) to precipitate nucleic acids and then fractionated by ammonium sulfate to remove excess PEI. The pellet was resolubilized and applied to Ni-NTA resin. The resulting eluate was further fractionated by size‐exclusion chromatography to yield a monodispersed NS preparation (Fig. 2A, panels iv and v). Mass spectrometry confirmed NS identity, with 95% sequence coverage and 147 unique peptides (Fig. 2B), while SDS–PAGE revealed ∼95% purity and the absence of detectable nuclease contaminants, validating the identity and purity of the recombinant NS protein for downstream biochemical and functional assays.

### NS is a *bona fide* DNA-binding protein

Our SIRF assays (Fig. 1A) had revealed that NS localizes to the nucleus and associates with nascent DNA. To determine if NS binds DNA directly, we performed electrophoretic mobility shift assays using the purified recombinant NS and a panel of replication-relevant substrates, including ssDNA, dsDNA, 5′-overhang and 3′-overhang DNA, and a reversed fork-mimicking DNA. Our results show that NS bound all of those substrates in a dose-dependent manner, displaying a moderate preference for ssDNA and the 5′- and 3′-overhang DNA molecules (Fig. 2C).

### NS protects reversed forks from MRE11-mediated degradation

**Figure 3.**
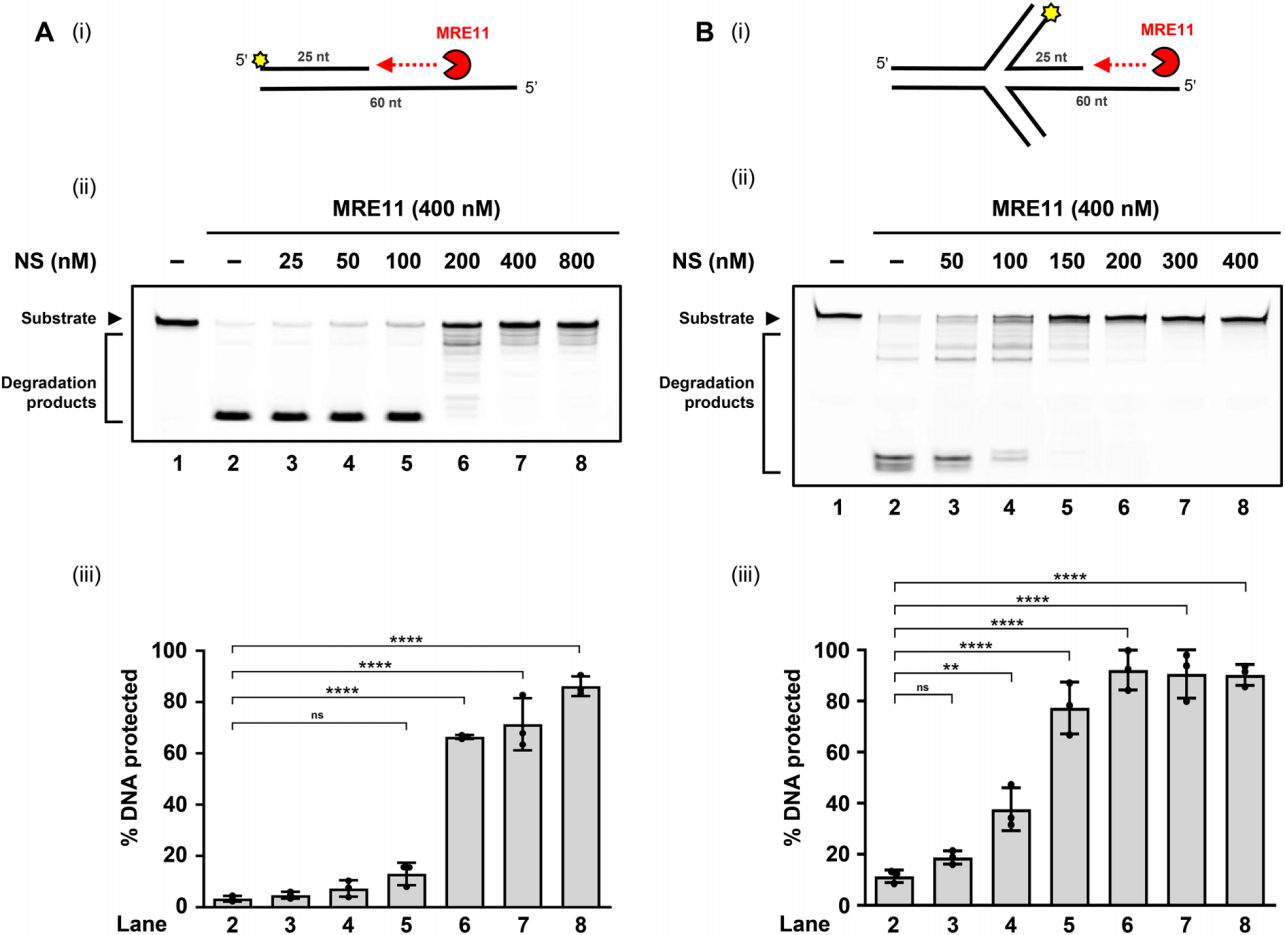
NS protects fork-mimetic substrates from MRE11-mediated degradation. (**A**) MRE11 protection assay using a 5′-overhang substrate. (i) A schematic of the Cy3-labeled 5′-overhang DNA substrate designed to mimic the regression arm of a reversed fork. (ii) Purified NS was preincubated with 58 nM substrate at 37°C for 10 min, then challenged with MRE11 for 40 min. DNA products were separated by 27% denaturing PAGE and visualized. (iii) Quantification of protected DNA from three independent experiments (mean ± SD); ns (not significant): *P* = 0.2132; ^****^P <0.0001. (**B**) MRE11 protection assay using a reversed-fork substrate. (i) A schematic of the Cy3-labeled four-way junction substrate. (ii) NS was incubated with 29 nM substrate at 37°C for 10 min, followed by MRE11 digestion for 80 min. DNA products were analyzed as in (**A**). (iii) Quantification of protected DNA from three independent experiments (mean ± SD); ns: *P* = 0.8533; **P = 0.0064; ^****^P <0.0001. All statistical values were determined by one-way ANOVA with Tukey’s post hoc test.

The ability of Mirin to rescue nascent‐strand loss in NS‐depleted cells underscores the function of NS as a fork protector under replication stress. To test this capability directly, we performed *in vitro* protection assays using synthetic 5′‐overhang substrates that mimic the regressed arm of a reversed fork (Fig. 3A, panel i). A high NS concentration (400 nM) preserved >70% of the substrate (Fig. 3A, panels ii and iii; lanes 2 versus 7), whereas a low concentration (≤100 nM) conferred minimal protection (lanes 3–5). Comparable results were obtained using a four-way junction substrate that more closely resembles a reversed fork (Fig. 3B). These findings demonstrate that NS directly shields reversed forks from MRE11-dependent nucleolytic degradation. Accordingly, we reasoned that NS might protect stalled forks by limiting MRE11 access to nascent DNA. To test this possibility, we assessed the association of MRE11 with nascent DNA. SIRF analysis revealed that NS depletion significantly increased the percentage of cells containing >10 MRE11-biotin PLA foci following HU treatment (Supplementary Fig. S3G–I), demonstrating that NS functions to restrain MRE11 access to stalled replication forks.

### Functional synergy of NS and RAD51 in protecting reversed replication forks

**Figure 4.**
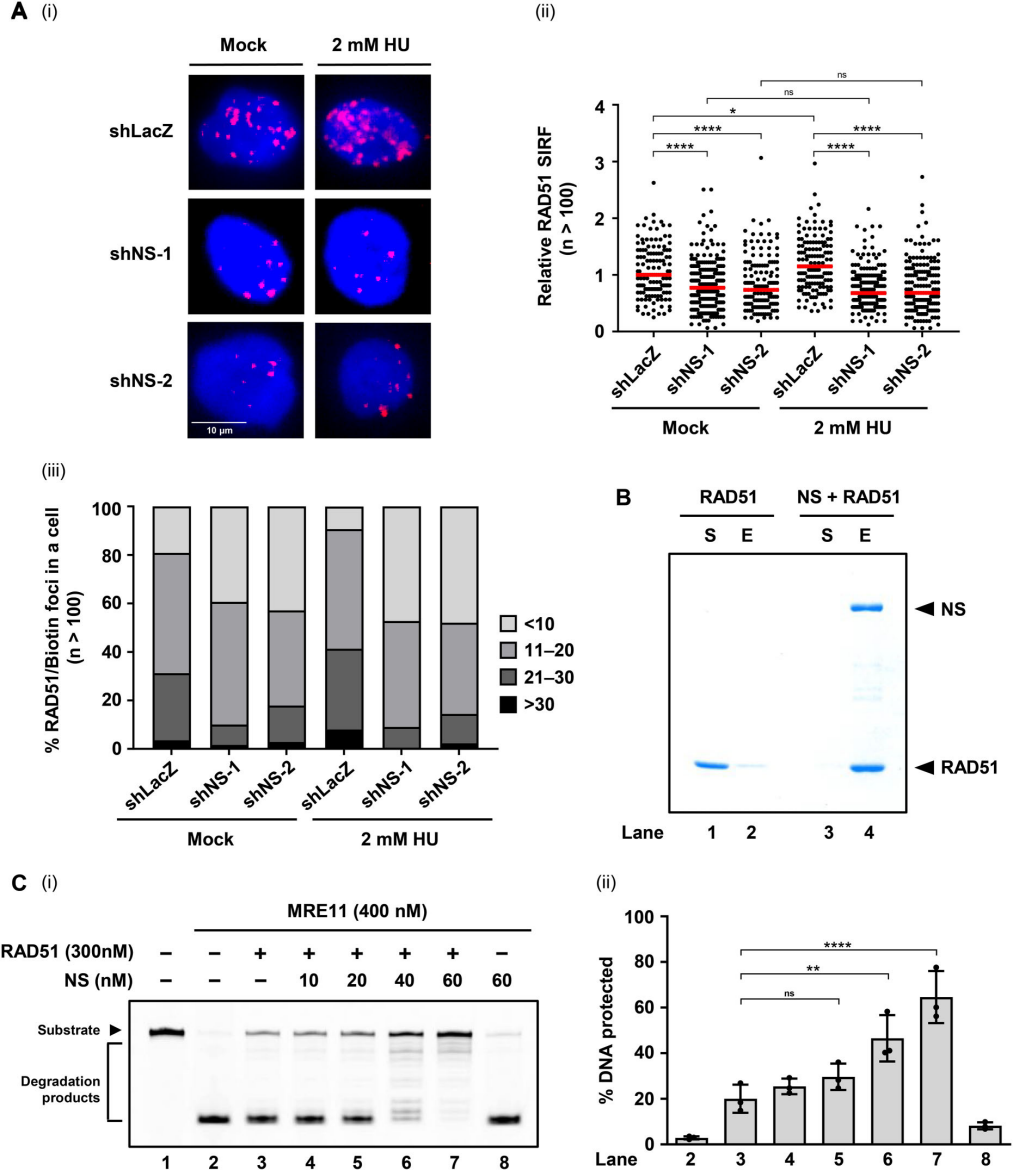
NS and RAD51 functionally cooperate at stalled forks. (**A**) Single-cell SIRF-based quantification of RAD51 at stalled forks. EdU-labeled nascent DNA was biotinylated via click chemistry, probed with anti-biotin and anti-RAD51 antibodies, and then visualized by proximity ligation. (i) Representative images of RAD51-biotin PLA foci (red) and Hoechst-stained nuclei (blue) in cells treated with or without 2 mM HU for 1 h in the control (shLacZ) or NS-depleted backgrounds (shNS-1 and shNS-2). (ii) The number of PLA foci per nucleus was determined from >100 cells for each condition. Relative SIRF was calculated by normalizing the number of PLA foci per cell to the median Alexa Fluor 488 signal of the corresponding sample and then to untreated cells. Statistical significance was determined by Mann–Whitney test. The *P*-value between Mock and HU treatment of shNS-1 groups is ns: *P* = 0.1496; and for the shNS-2 groups it is ns: *P* = 0.5418; *P = 0.0127; ^****^P <0.0001. The red line indicates the median. (iii) The number of PLA foci per nucleus was binned into four categories (<10, 11–20, 21–30, >30), and the percentage of cells in each category is plotted. (**B**) Direct protein–protein interaction between NS and RAD51 was assessed by affinity pull-down assay. Purified NS and RAD51 were incubated together and captured on His-Tag Dynabeads. Supernatant (S) and eluted (E) fractions were resolved by 10% SDS–PAGE and stained with Coomassie Blue. (**C**) MRE11 protection assay. The Cy3-labeled 5′-overhang substrate was first incubated with RAD51, then with NS, before challenge with MRE11 for 40 min. Protection efficiency was quantified from three independent experiments (mean ± SD) and analyzed by one-way ANOVA with Tukey’s post hoc test; ns: *P* = 0.6066; **P = 0.004; ^****^P <0.0001.

RAD51 has been shown to safeguard reversed forks from MRE11-mediated degradation by forming nucleoprotein filaments on the nascent DNA [23, 24]. Given that NS depletion markedly reduces the generation of RAD51 foci under HU treatment [40], we investigated if NS is required for RAD51 localization at stalled forks. To do so, we depleted NS via two independent lentiviral shRNAs (Fig. 1B, panel iii). Subsequent SIRF assays demonstrated RAD51 enrichment at stalled forks following HU treatment in control cells, but this enrichment was significantly attenuated in the NS-deficient cells (Fig. 4A, panel i and ii). Under normal growth conditions, ∼30% of shLacZ control cells displayed >20 RAD51-biotin PLA foci, whereas NS KD reduced this percentage to ∼10% (Fig. 4A, panel iii). Upon HU exposure, ∼40% of shLacZ control cells exhibited >20 RAD51-biotin PLA foci, compared to only 10%–15% of NS-depleted cells that did so (Fig. 4A, panel iii). These data establish that NS is essential for efficient RAD51 localization at stalled replication forks. To further validate the effect of NS depletion on RAD51 accumulation during replication stress, we examined RAD51 foci formation in S-phase cells following HU treatment. To address this, cells were pulse-labeled with EdU and subsequently treated with 2 mM HU for 5 or 24 h. EdU was then conjugated to Alexa Fluor 488 via click chemistry to identify S-phase cells. Analysis of Alexa Fluor 488-positive cells demonstrated a time-dependent increase in RAD51 foci following HU treatment (Supplementary Fig. S4). Importantly, NS depletion significantly impaired RAD51 foci formation, reducing the fraction of cells containing > 5 RAD51 foci by approximately two-fold at both time points (Supplementary Fig. S4C). Together, these data demonstrate that NS promotes RAD51 accumulation at stalled replication forks, highlighting its critical role in fork protection during replicative stress.

To establish a direct interaction between NS and RAD51, we performed an *in vitro* pull-down assay, whereby purified MBP-NS-His₆ was immobilized on Co²⁺ Dynabeads to pull down purified RAD51. Our results show that RAD51 co-eluted with MBP-NS-His₆ but not with control Dynabeads (Fig. 4B), confirming a direct protein–protein interaction. Next, we examined the functional synergy of NS and RAD51. In an MRE11 nuclease protection assay, RAD51 alone preserved ∼20% of a 5′-overhang substrate (Fig. 4C, lane 3), whereas addition of 60 nM NS enhanced protection approximately three-fold (to ∼60%) (lane 7). A similar cooperative effect was observed when we used a reversed fork-mimicking DNA substrate. In that case, NS or RAD51 individually afforded limited protection, but together they markedly suppressed MRE11-mediated degradation (Supplementary Fig. S5A and B). Additionally, we used *E. coli* RecA at the same concentration as human RAD51 in the protection assay to test whether NS could confer a cooperative protective effect with RAD51 ortholog. Our results indicate no cooperativity between NS and RecA in fork protection, providing evidence for the species specificity of synergistic NS-RAD51 activity in DNA protection (Supplementary Fig. S5C and D). These findings demonstrate that NS facilitates RAD51 localization at stalled forks and acts in concert with RAD51 to protect reversed forks against MRE11-mediated nucleolytic attack.

### RAD51 nucleoprotein filament is essential for NS-RAD51 synergy

**Figure 5.**
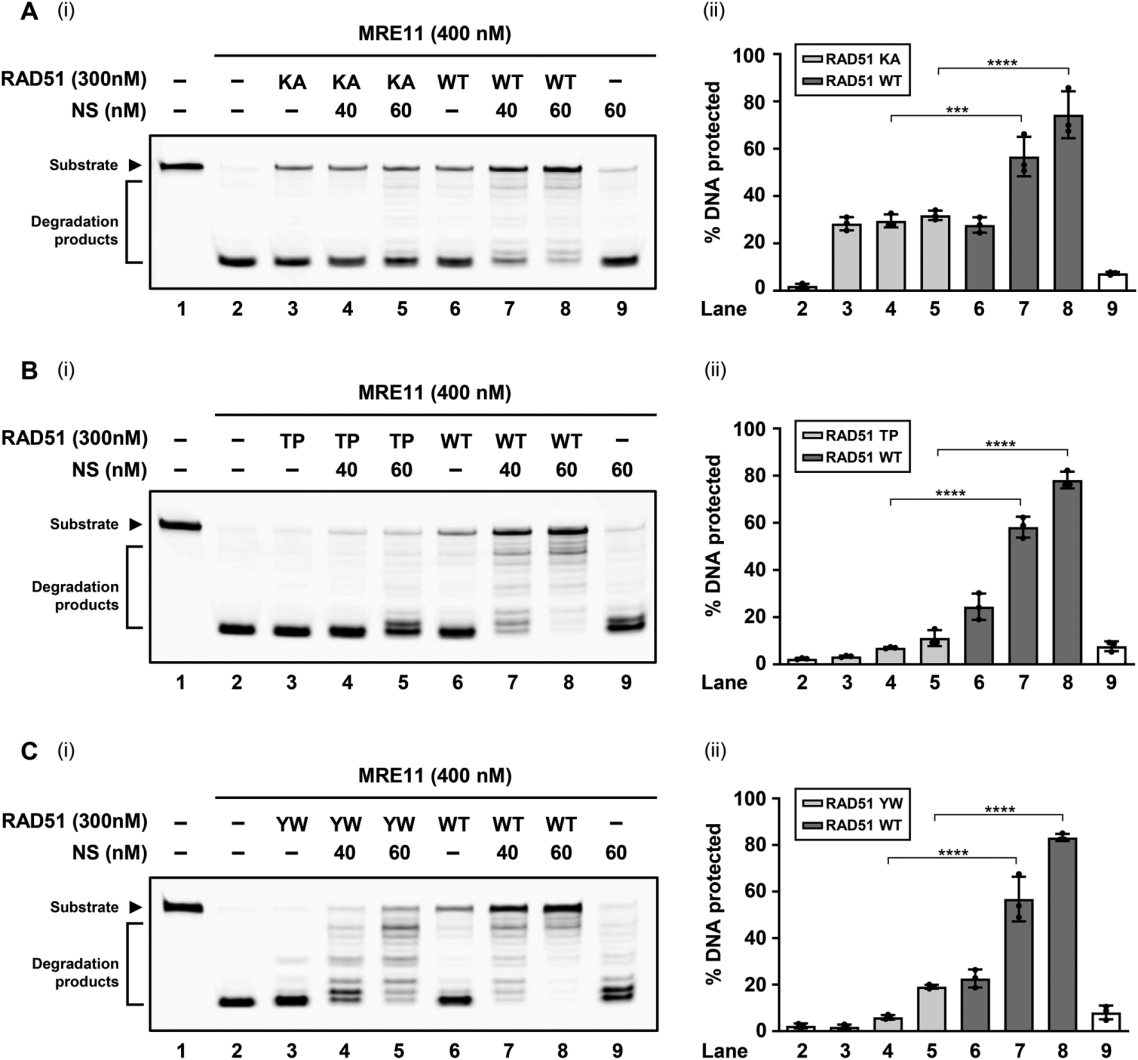
A functional RAD51 filament is essential for synergistic NS- and RAD51-mediated DNA protection. MRE11 protection assay using a Cy3-labeled 5′-overhang substrate. The substrate was first incubated with wild-type RAD51 (WT) or one of three mutants—(**A**) K133A (KA), (**B**) T131P (TP), or (**C**) Y232W (YW)—followed by incubation with NS and then challenged with MRE11. DNA products were separated by 27% denaturing PAGE and visualized. Note that 0.1 mM ATP was used in the K133A reaction to ensure the RAD51 KA variant lacked ATP-binding capability. (i) Representative gel image. (ii) Quantification of protected DNA from three independent experiments (mean ± SD). Statistical significance was determined by one-way ANOVA with Tukey’s post hoc test; ***P = 0.0001; ^****^P <0.0001.

To further delineate the mechanism by which NS and RAD51 cooperate in fork protection, we determined which of RAD51’s functions are required for NS-RAD51 synergy. First, we tested the synergy between NS and an ATP-binding-deficient RAD51 K133A mutant, as ATP binding is required for RAD51 to form protective nucleoprotein filaments [25, 47]. Although the K133A mutant interacted with NS as effectively as wild-type RAD51 (Supplementary Fig. S6), it failed to enhance NS-mediated DNA protection (Fig. 5A). This result demonstrates that functional nucleoprotein filament formation is indispensable for the cooperative effect of NS and RAD51. Next, we evaluated the synergy between NS and two DNA-binding-defective RAD51 variants. The clinical Fanconi anemia-linked T131P mutant, which promotes fork reversal but not protection [24, 30, 31], markedly reduced NS synergy in nuclease protection assays (Fig. 5B). Likewise, the Y232W mutant—defective in loop-1 DNA binding [56]—showed minimal cooperation with NS (Fig. 5C). Importantly, both mutant variants retained NS interaction (Supplementary Fig. S6). These results demonstrate that both the DNA-binding and functional filament assembly of RAD51 are prerequisites for the NS-RAD51 complex to protect reversed forks synergistically from MRE11-mediated degradation.

### NS promotes the assembly of RAD51 nucleoprotein filaments

**Figure 6.**
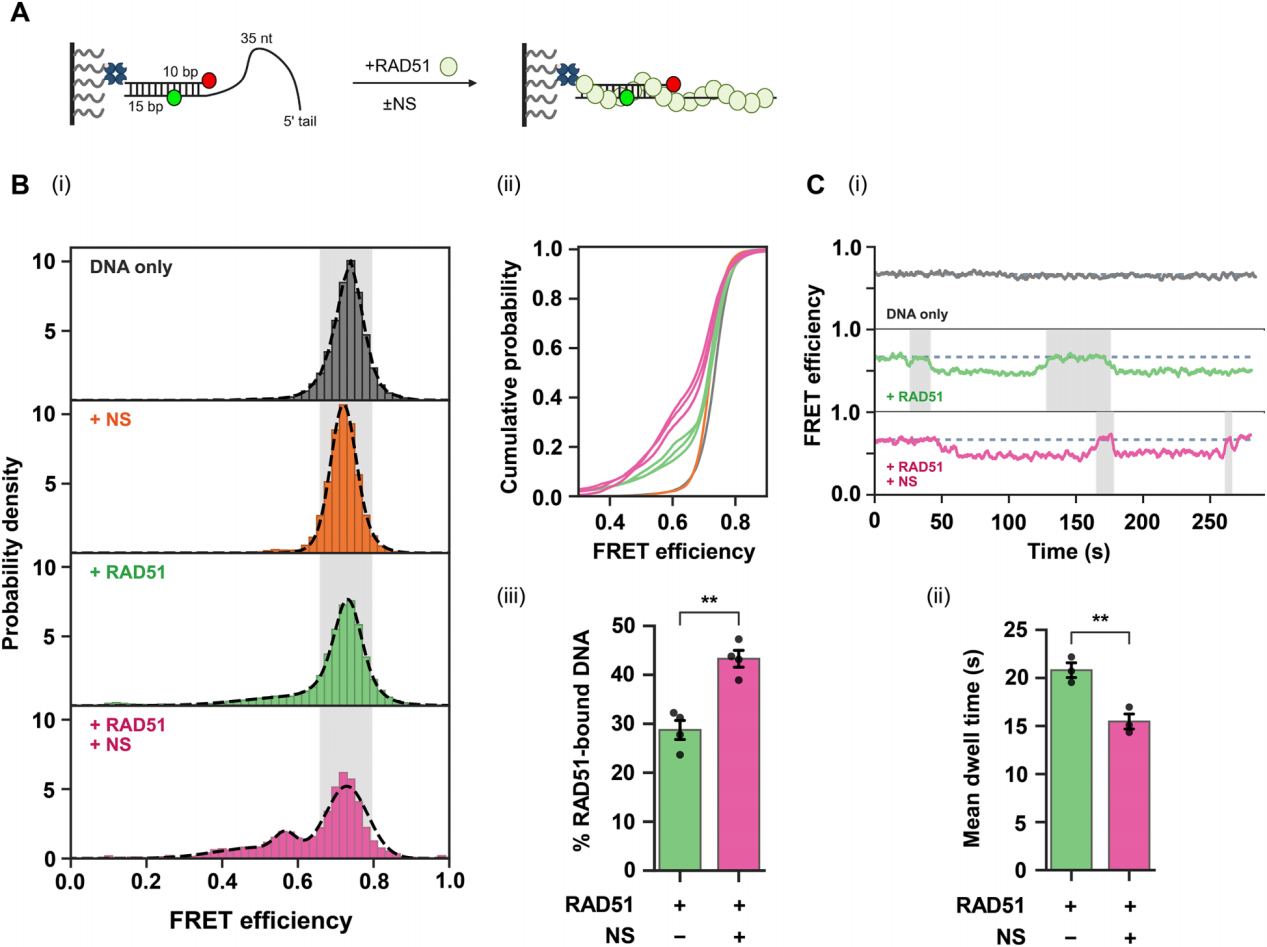
NS recruits RAD51 to dsDNA and enhances RAD51 nucleoprotein filament formation. (**A**) A schematic of the RAD51 assembly smFRET assay on the DNA substrate with dye pairs located at the double-stranded segment near the ssDNA/dsDNA junction. (**B**) (i) FRET histograms of DNA only, with NS alone, RAD51 alone, or a RAD51-NS mixture. The gray-shaded area represents the DNA-only state, and its FRET is ∼0.727 ± 0.068. (ii) The cumulative FRET probabilities from three independent experiments were plotted as unbinned empirical cumulative densities, with the same color scheme as used in the histograms (panel i). (iii) The RAD51-bound fraction of FRET DNA substrates with dye pairs located at the double-stranded region was derived from the probabilities lower than the left bound of the gray-shaded DNA-only area in (i), and quantified from three independent experiments with >250 total trajectories for each condition [mean ± standard error of the mean (SEM)]; **P = 0.0014. (**C**) (i) Representative smFRET time-courses of DNA only, with RAD51 or a RAD51-NS mixture. Dwell times of the FRET ∼0.7 state (DNA-only state) are highlighted and shaded gray. (ii) Mean dwell times of the FRET ∼0.7 state were quantified from three independent experiments with >200 total trajectories for each condition (mean ± SEM); **P = 0.0082. All statistical significance values were determined by Student’s *t*-test.

The binding of RAD51 to dsDNA has been documented previously for its protective effect against MRE11-mediated DNA degradation [57]. To elucidate how NS influences RAD51 filament assembly mechanistically, we employed a smFRET assay, which is a sensitive means of monitoring nucleoprotein filament assembly. When recombinases bind to DNA substrates containing the FRET dye pair, they lengthen the DNA, increasing dye pair separation and resulting in a lower FRET efficiency [58]. First, we used a DNA substrate containing a 35-nucleotide (nt) 5′ single-stranded overhang, with the Cy3/Cy5 FRET dye pair located at the dsDNA segment near the ssDNA/dsDNA junction (Fig. 6A, for the schematic diagram), the entry point of MRE11. This DNA substrate was incubated with RAD51 in the presence or absence of NS to monitor RAD51 binding. As expected, RAD51 binding resulted in an increase in the lower FRET population compared to the DNA substrate alone control (Fig. 6B, panel i, green versus gray), whereas NS alone did not alter FRET (Fig. 6B, panel i, orange). Notably, in the mixture of RAD51 and NS, we detected a significantly enhanced probability density for the low-FRET condition (Fig. 6B, panel i, pink versus green), corresponding to an increase in RAD51-bound DNA. This outcome can be clearly interpreted from the cumulative probability plot, in which a higher cumulative probability is observed at lower FRET for the RAD51-NS mixture (Fig. 6B, panel ii, pink curve). Quantitative analyses revealed that the fraction of RAD51-bound DNA increased by >10% in the presence of NS (Fig. 6B, panel iii). Interestingly, similar NS stimulation of the RAD51-bound fraction can be observed for the FRET DNA substrate with a dye pair located at the distal double-stranded segment further from the junction (Supplementary Fig. S7A). Taken together, these results demonstrate that NS stimulates RAD51 filament assembly on the dsDNA region near the ssDNA/dsDNA junction.

Next, we examined if NS enhances RAD51 binding to ssDNA by positioning the Cy3/Cy5 FRET dye pair to the ssDNA segment near the ssDNA/dsDNA junction (Supplementary Fig. S7B, panel i). Surprisingly, in the presence of the RAD51-NS mixture, no apparent stimulation of the RAD51-bound population was observed (Supplementary Fig. S7B, panels ii–iv), indicating that NS promotes RAD51 filament assembly on dsDNA but not on ssDNA, thereby suppressing MRE11 access to the duplex region through the ssDNA/dsDNA junction.

### NS facilitates RAD51 loading onto dsDNA

To gain further insights into the mechanism by which NS promotes RAD51 filament assembly, we analyzed real-time FRET time-courses using a hidden Markov model (HMM) [59], which has been deployed previously to identify states associated with dynamic conversion and helped to elucidate how other accessory proteins stimulate recombinase assembly [49, 50, 60]. Representative FRET time-courses are shown in Fig. 6C (panel i), revealing apparent FRET alternations indicative of dynamic RAD51 binding to and dissociation from DNA substrates in real-time. A clustered transition density plot of FRET transitions validated the HMM state assignment (Supplementary Fig. S8). Here, we focused on the ∼0.7 FRET state, the DNA-only state, with the lower FRET states (<0.7) indicating RAD51-bound states. Accordingly, the dwell time of the 0.7 FRET state reflects the association rate of RAD51 on the dsDNA segment, with a shorter dwell time indicating a faster association rate. Our results show that NS shortened the dwell time of the 0.7 FRET state (Fig. 6C, panel ii), implying enhanced RAD51 binding to the dsDNA segment. A control experiment using the DNA substrate with dyes in the ssDNA region showed no difference in dwell time in the presence or absence of NS (Supplementary Fig. S9). These results demonstrate that NS specifically promotes RAD51 loading on dsDNA near the ssDNA/dsDNA junction by preferentially enhancing RAD51 binding, thereby promoting protection against MRE11-mediated degradation.

## Discussion

A recent study proposed that NS prevents nascent strand degradation via an indirect mechanism of CDC7 inhibition, which limits excessive replication origin firing [44]. However, this model does not address the direct role of NS in protecting stalled replication forks as evidenced by its interaction with RAD51. In this study, we present multiple lines of evidence showing that NS directly accumulates at HU‐stalled forks in cells, binds fork‐mimicking DNA *in vitro*, and partners with RAD51 to form a protective complex that blocks MRE11-mediated degradation. Together with the study by Lebdy *et al*. [44], these findings support a two-phase model whereby NS first acts in early S phase within the nucleolus to restrain origin firing by sequestering ORC2. Upon replication stress, NS becomes chromatin-bound and shifts its role to synergize with RAD51 in stabilizing reversed forks. These sequential functions reveal NS as a multifaceted regulator of genome integrity that acts both upstream to control replication initiation and downstream to preserve fork stability.

Our biochemical analyses demonstrate that NS protects reversed forks via two complementary and concentration-dependent mechanisms. At high concentrations, the intrinsic DNA-binding activity of NS allows it to protect the regressed arm of reversed forks, similar to the mechanism by which the FANCD2-FANCI complex binds DNA and physically blocks MRE11 entry [61]. In this mode, abundant NS forms a protein barrier that directly shields nascent strands from nuclease attack. At low concentrations, NS functions as an auxiliary factor by recruiting RAD51 to stalled forks and promoting nucleoprotein filament assembly for fork protection. The cooperative action of NS and RAD51 in replication fork protection may be explained by two nonmutually exclusive mechanisms. First, the preferential binding of NS to ssDNA could indirectly promote RAD51 accumulation on dsDNA by limiting RAD51 access to ssDNA. Alternatively, NS may directly facilitate RAD51 filament assembly on dsDNA, thereby promoting the formation of a protective RAD51 nucleoprotein filament at the ssDNA/dsDNA junction. Our data favor the latter model. Single-molecule FRET analyses demonstrated that NS selectively enhances RAD51 loading onto the dsDNA region adjacent to the ssDNA/dsDNA junction (Fig. 6), whereas it has no detectable effect on RAD51 occupancy or binding dynamics on ssDNA (Supplementary Figs S7B and S9). These observations support a model in which NS directly promotes RAD51 filament assembly on dsDNA, thereby contributing to the protection of stalled replication forks from nuclease-mediated degradation. Our observation aligns with the notion that the dsDNA-binding capacity of RAD51 evolved to support fork protection and mirrors the DNA-protection role of BRCA2 [57]. Importantly, we show herein that RAD51 filament assembly is essential for NS-RAD51 synergetic action, as NS cannot protect DNA with RAD51 mutants defective in forming nucleoprotein filaments. Together, our findings underscore the dual necessity of direct DNA occupancy by NS and stable RAD51 nucleoprotein filaments for safeguarding reversed forks. To further dissect the interdependence of these two activities of NS in conferring its fork protection function, separation-of-function NS mutants selectively defective in either DNA binding or RAD51 interaction will be required.

NS is predominantly concentrated in the nucleolus, the site of ribosome biogenesis [32]. This organelle forms around tandem repeats of ribosomal DNA (rDNA) on acrocentric chromosomes. Its high transcriptional activity and repetitive structure make it particularly vulnerable to DSBs and replication–transcription conflicts [62, 63]. Our findings indicate that nucleolar NS may play a specialized role in safeguarding the integrity of rDNA. By binding stalled replication forks and antagonizing MRE11, NS may protect nascent rDNA strands from nuclease attack during intense transcription or replication stress. RAD51 is also known to contribute to rDNA maintenance [64, 65]. Our finding that NS and RAD51 act in concert to protect reversed forks points to a similar partnership within the nucleolus. In this scenario, NS promotes RAD51 filament assembly at damaged rDNA or stalled forks, facilitating efficient repair while limiting unnecessary resection. Such cooperation would reinforce nucleolar resilience against genotoxic insults, preserve rDNA copy number, and sustain ribosome production under adverse conditions. In addition to supporting the maintenance of highly vulnerable rDNA loci, nucleolar localization of NS may provide a regulatory storage or sequestration mechanism that spatially controls its fork-protective activity. NS may be retained in the nucleolus under basal conditions, and released to the nucleoplasm upon replication stress to support RAD51-mediated fork protection. Such spatial regulation could help prevent inappropriate RAD51 association with dsDNA while preserving its primary role in ssDNA binding during homologous recombination. Future investigations should examine the colocalization of NS and RAD51 at rDNA and assess how NS depletion affects rDNA stability.

## Supplementary Material

gkag773_Supplemental_File

## Data Availability

The data underlying this article are available in the article and in its online Supplementary Material.
