# Supplementary material for: Nucleostemin promotes RAD51 filament assembly on double-stranded DNA to protect stalled replication forks
Source: Nucleic Acids Res. 2026 Aug 10;54(15):gkag773. doi: 10.1093/nar/gkag773 (PMC13454837; doi:10.1093/nar/gkag773)
Supplement: Supplementary file 1 [file gkag773_supplemental_file.pdf]

Supplementary Information

Nucleostemin promotes RAD51 filament assembly on double-stranded DNA to protect stalled replication forks

Chih-Chun Chang[1#], Siang-Sheng Tsai[2#], Chang-Le Chou[3,4], Yi-Hsuan Chang[1], Chia-Tai Hung[2], Robert Y. L. Tsai[5,6], Hung-Wen Li[3*], Hungjiun Liaw[2*], and Peter Chi[1,7*]

[1]Institute of Biochemical Sciences, National Taiwan University, Taipei, Taiwan

[2]Department of Life Sciences, National Cheng Kung University, Tainan, Taiwan

[3]Department of Chemistry, National Taiwan University, Taipei, Taiwan

[4]Department of Pharmacy, National Taiwan University, Taipei, Taiwan

[5]Institute of Biosciences and Technology, Texas A&M University Health Science Center, Houston, Texas, USA

[6]Department of Translational Medical Sciences, College of Medicine, Texas A&M University Health Science Center, Houston, Texas, USA.

[7]Institute of Biological Chemistry, Academia Sinica, Taipei, Taiwan

# Both authors contributed equally to this work.

[*] Correspondence: peterhchi@ntu.edu.tw; liawh@mail.ncku.edu.tw; hwli@ntu.edu.tw

This PDF file includes:

Supplementary Figures S1 to S10

Supplementary Tables S1 to S3

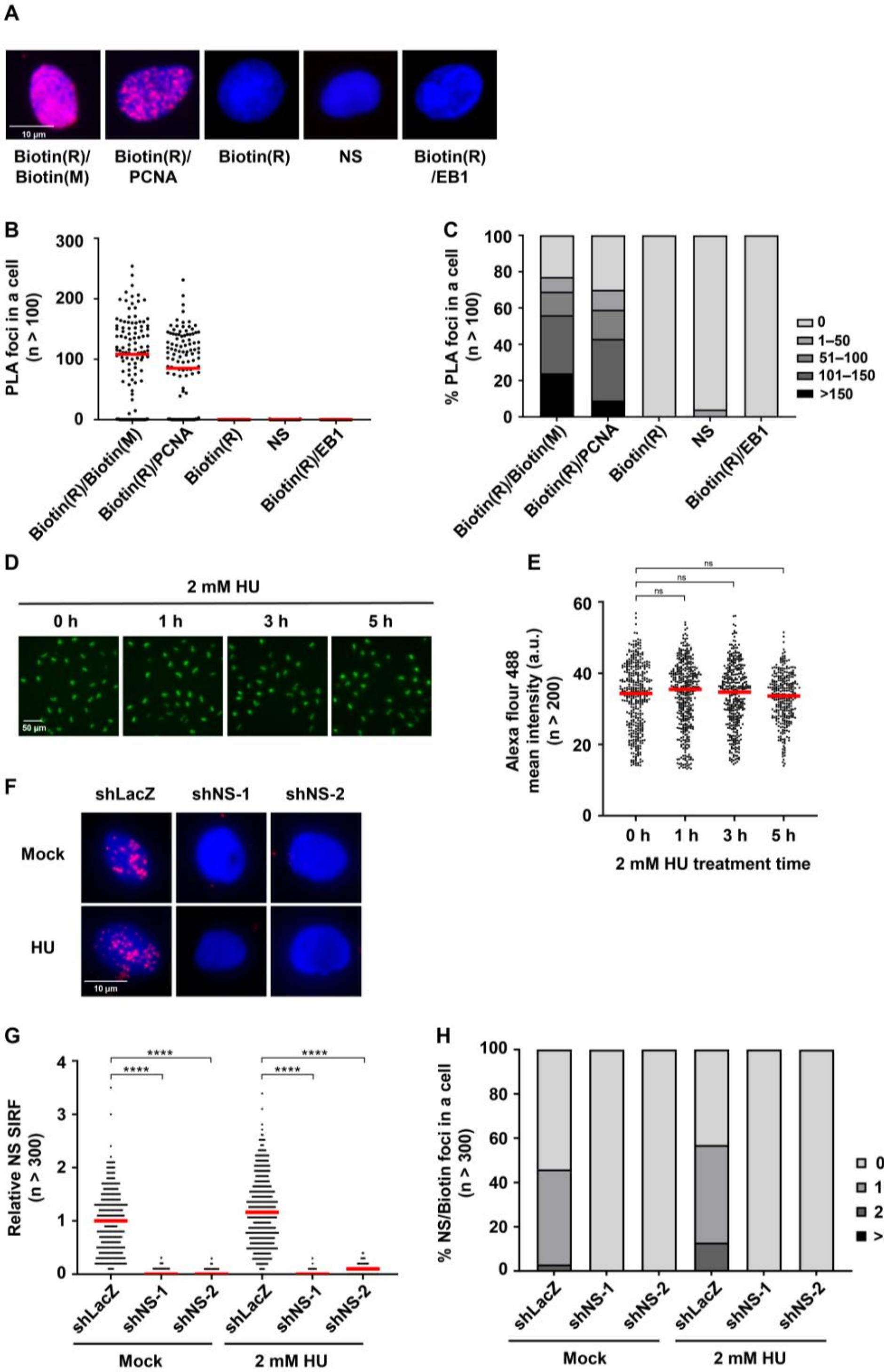

Supplementary Figure S1. (**A-C**) Validation of the SIRF assay. Positive controls included a combination of rabbit and mouse anti-biotin antibodies (Biotin R/Biotin M) and rabbit anti-biotin with mouse anti-PCNA (Biotin R/PCNA). Negative controls comprised a single anti-biotin antibody (Biotin R), a single anti-NS antibody (NS), and rabbit anti-biotin with mouse anti-EB1 (Biotin R/EB1). (**A**) Representative PLA images. Red dots indicate PLA foci; nuclei are stained with Hoechst 33258 (blue). Antibody combinations are labeled. (**B**) PLA foci per nucleus was quantified from >100 cells for each condition. The red line indicates the median. (**C**) The number of PLA foci was

binned into five categories (0, 1–50, 51–100, 101–150, >150), and the percentage of cells in each bin is plotted. (**D**-**E**) Assessment of EdU incorporation under replication stress. **(D)** Representative images of Alexa Fluor 488 signals (green) showing EdU incorporation in cells treated with or without 2 mM HU for 0, 1, 3, or 5h. **(E)** Alexa Fluor 488 intensity per nucleus was quantified from >200 cells per condition. Statistical significance was determined by the Mann-Whitney test; ns, not significant. The red line indicates the median. (**F**-**H**) NS-SIRF analysis following NS depletion. **(F)** Representative SIRF images of NS-biotin PLA foci (red) and Hoechst-stained nuclei (blue) in NS-depleted cells following treatment with or without 2 mM HU for 1h. **(G)** PLA foci per nucleus were quantified from >300 cells for each condition. Relative NS SIRF was calculated by normalizing the number of NS-biotin PLA foci per cell to the median of their respective Alexa-488 signals and to untreated shLacZ cells. Statistical significance was determined by the Mann-Whitney test. The *P* values for the mock and HU treatments are ****$P < 0.0001$. The red line indicates the median. **(H)** The number of PLA foci per nucleus was binned into four categories (0–10, 11–20, 21–30, >30 foci), and the percentage of cells in each category is plotted.

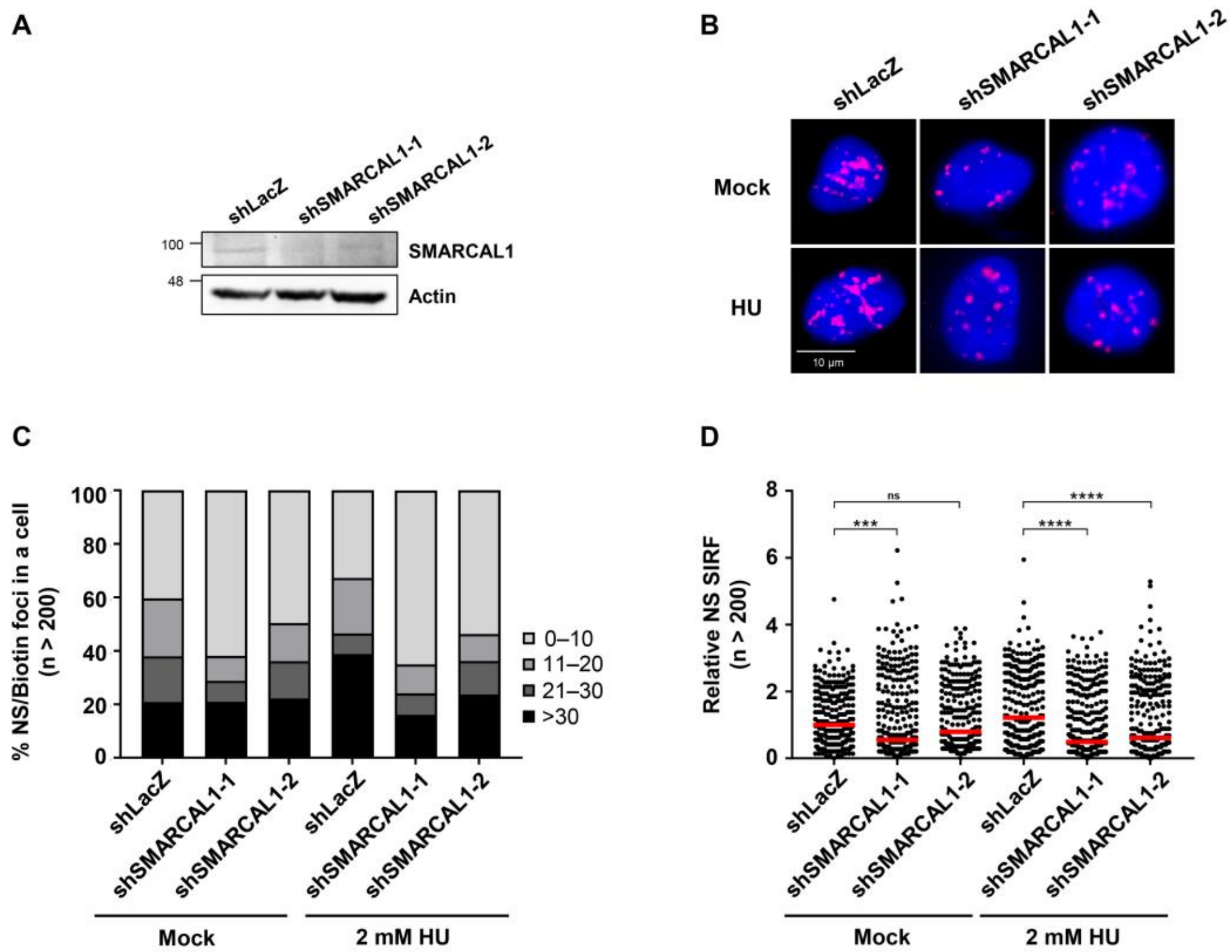

Supplementary Figure S2. SMARCAL1 depletion impairs NS recruitment to stalled replication forks. (**A**) Western blot validation of SMARCAL1 knockdown in T24 cells using two distinct shRNAs (shSMARCAL1-1 and shSMARCAL1-2). (**B**) Single-cell SIRF-based quantification of NS at stalled forks. Representative images of NS-biotin PLA foci (red) and Hoechst-stained nuclei (blue) in shLacZ or shSMARCAL1 cells treated with or without 2 mM HU for 1 h. (**C**) The number of PLA foci per nucleus was binned into four categories (0–10, 11–20, 21–30, >30 foci), and the percentage of cells in each category is plotted. (**D**) PLA foci per nucleus was quantified from >200 cells per condition. Relative NS-SIRF was calculated by normalizing the number of NS-biotin PLA foci per cell to the median Alexa Fluor 488 signal of the corresponding sample and then to untreated cells. Statistical significance was determined by the Mann-Whitney test; ns (not significant): $P > 0.05$; $***P = 0.0009$; $****P < 0.0001$. The red line indicates the median.

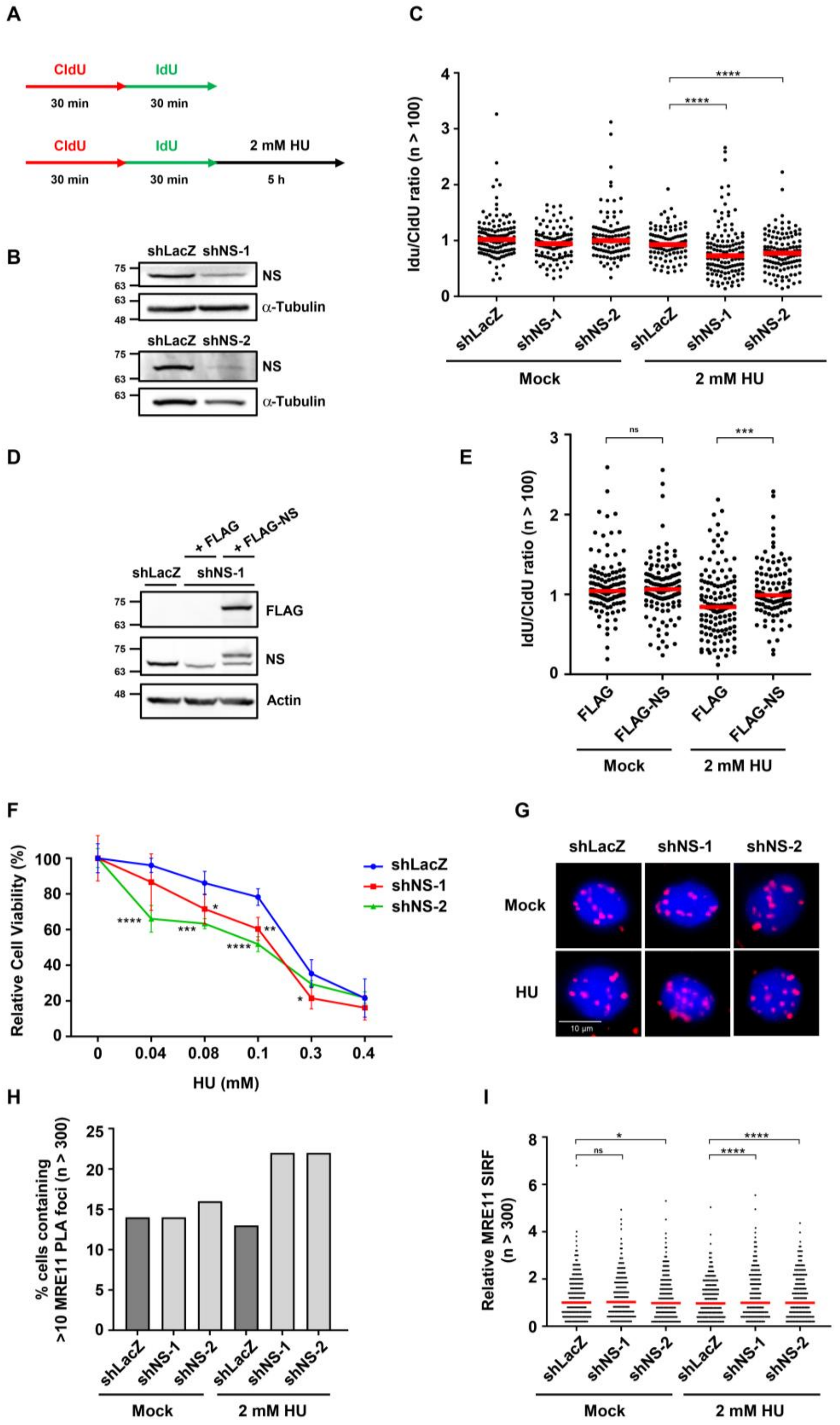

Supplementary Figure S3. NS protects reversed forks from MRE11-mediated degradation in U2OS cells. (**A**) A schematic of the DNA fiber assay design. U2OS cells in the wild-type or NS-knockdown (KD) backgrounds were sequentially labeled with CldU and IdU, then incubated ± 2 mM HU for 5 h. **(B**) Western blot of NS in control

(shLacZ) and NS-KD (shNS-1, shNS-2) cells. (**C**) Quantification of IdU:CldU tract-length ratios from >100 fibers per condition. Statistical significance was determined by Mann-Whitney test; ****P < 0.0001. **(D)** Western blots of FLAG and FLAG-NS in control (shLacZ) and shNS cells. When probed with anti-NS antibody, two bands were detected: the upper band represents FLAG-NS, whereas the lower band represents endogenous NS. **(E)** Fork stability during 5 h exposure to 2 mM HU. IdU:CldU ratios were quantified from >100 fibers. Statistical significance was determined using the Mann-Whitney test. The P value is ***P = 0.0002. The red line indicates the median. **(F)** MTT assay showing the viability of shLacZ control and NS-depleted cells following treatment with 0, 0.04, 0.08, 0.1, 0.3, or 0.4 mM HU for 5 days. Statistical significance was determined by the two-way ANOVA test. Quantification of cell viability from three independent experiments (mean ± SD). *P < 0.05; **P < 0.01; ***P < 0.001; ****P < 0.0001. **(G)** Single-cell SIRF-based quantification of MRE11 at stalled forks. Representative images of MRE11-biotin PLA foci (red) and Hoechst-stained nuclei (blue) in shLacZ or shNS cells treated with or without 2 mM HU for 1 h. **(H)** PLA foci per nucleus was quantified from >300 cells per condition. The percentage of cells containing more than 10 MRE11 foci was quantified and plotted. **(I)** Relative MRE11 SIRF was calculated by normalizing the number of MRE11-biotin PLA foci per cell to the median Alexa Fluor 488 signal of the corresponding sample and then to untreated cells. All statistical significance values were determined by Mann-Whitney tests; ns (not significant): P = 0.0807; *P = 0.0295; ****P < 0.0001. The red line indicates the median.

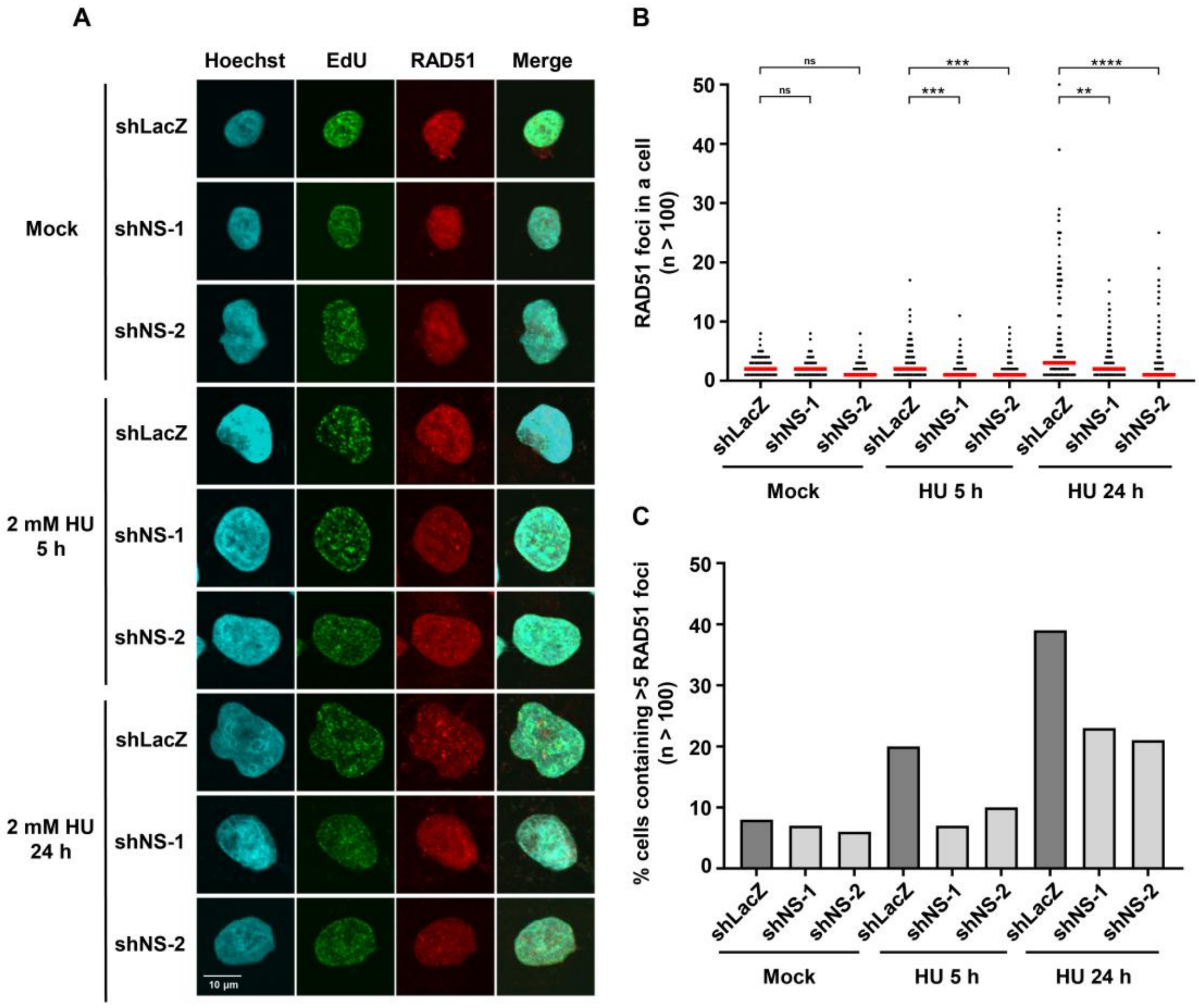

Supplementary Figure S4. NS depletion impairs RAD51 foci formation in S-phase cells following HU treatment. **(A)** Representative images of EdU (green), RAD51 (red), and Hoechst-stained nuclei (blue) in cells treated with or without 2 mM HU for 5 or 24 h. EdU was visualized by Alexa Fluor 488 conjugation via click chemistry. **(B)** RAD51 foci in cells during S-phase were quantified from >100 cells. Statistical significance was determined by the Mann-Whitney test. Under 5 h HU treatment, the *P* values for shNS-1 and shNS-2 versus shLacZ were ***$P = 0.0004$ and 0.0006, respectively. Under 24 h HU treatment, the *P* values for shNS-1 and shNS-2 versus shLacZ were **$P = 0.0010$ and ****$P < 0.0001$. The red line indicates the median. **(C)** The percentage of cells containing more than five RAD51 foci was quantified and plotted.

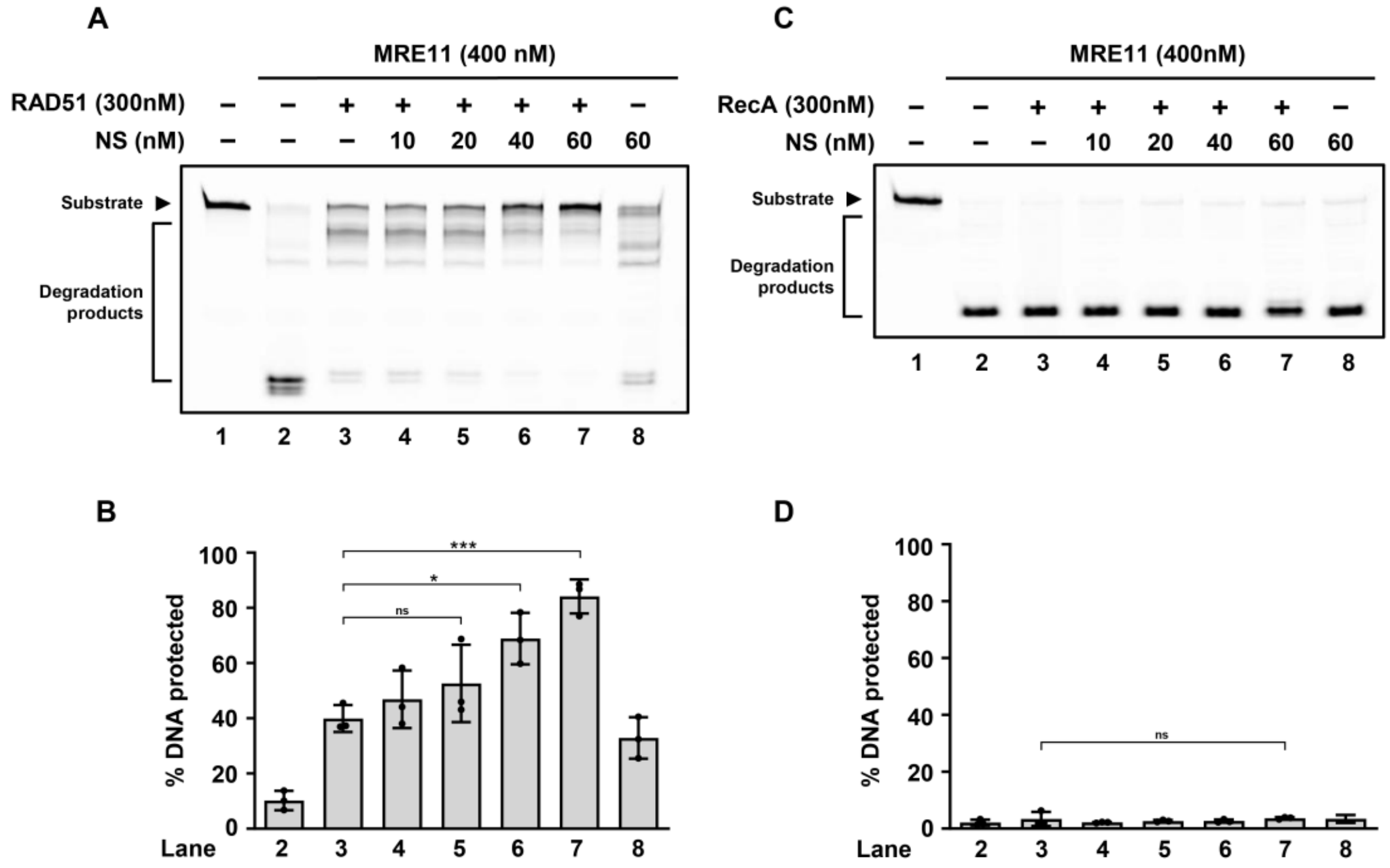

Supplementary Figure S5. NS and RAD51 synergistically protect reversed fork DNA. (**A**) Cy3-labeled reversed fork-mimicking DNA substrates were sequentially incubated with purified RAD51, followed by NS, and then challenged with MRE11 for 80 minutes. Reaction products were resolved by 27% denaturing PAGE and visualized. (**B**) Protection efficiency was quantified from three independent experiments (mean ± SD). Statistical significance was determined by one-way ANOVA with Tukey's post hoc test; ns (not significant): $P = 0.5692$; *$P = 0.0143$; ***$P = 0.0003$. **(C)** Cy3-labeled 5′-overhang substrates were sequentially incubated with RecA, followed by NS, and then challenged with MRE11 for 40 minutes. Reaction products were resolved by 27% denaturing PAGE and visualized. **(D)** Protection efficiency was quantified from three independent experiments (mean ± SD) and analyzed by one-way ANOVA with Tukey's post hoc test; ns: $P = 0.9999$.

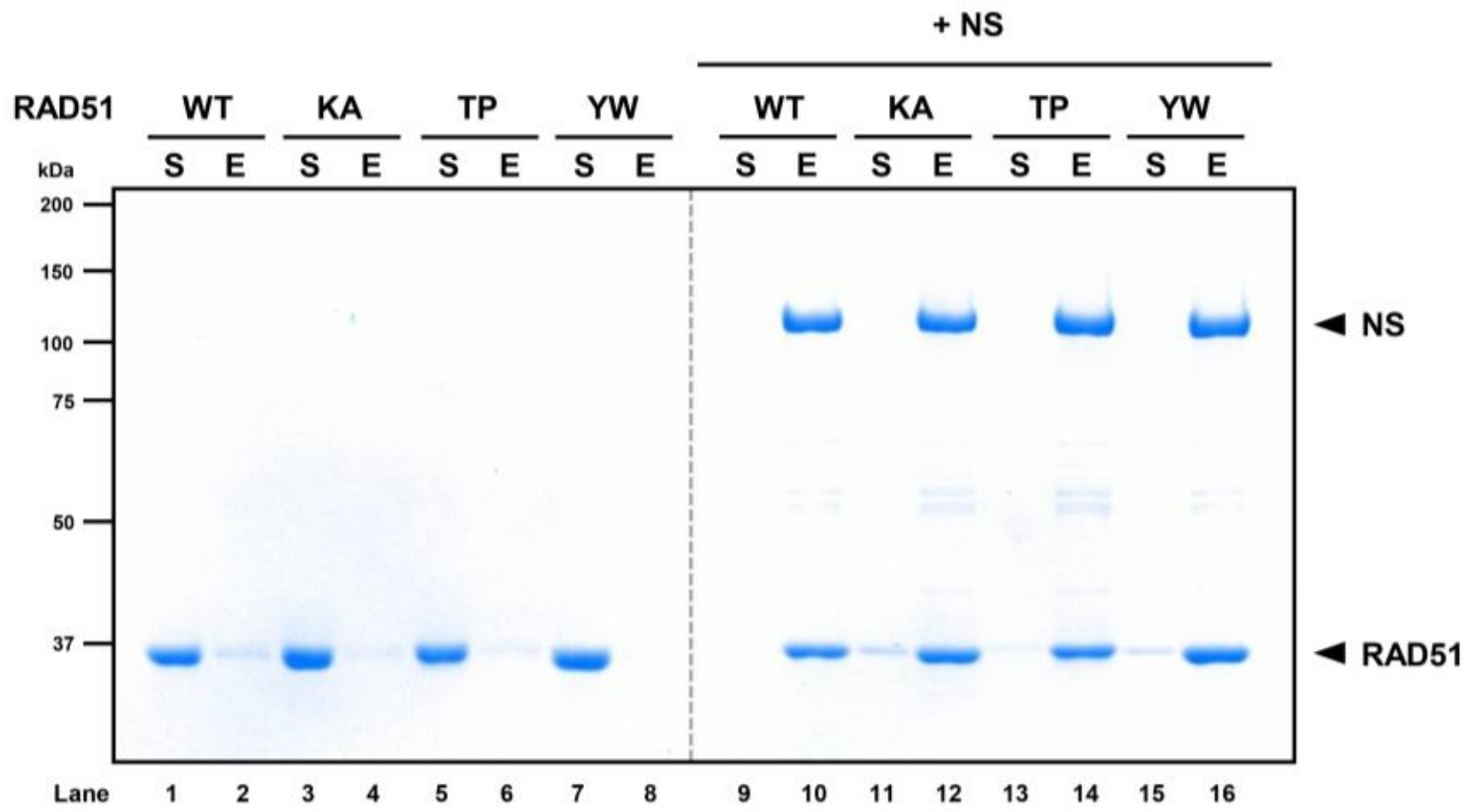

Supplementary Figure S6. NS physically interacts with RAD51 mutant variants. Direct protein-protein interactions between NS and RAD51 variants were examined by affinity pull-down using purified proteins. NS was incubated with wild-type RAD51 (WT) or the indicated RAD51 mutants—K133A (KA), T131P (TP), and Y232W (YW)—then captured using His-tag Dynabeads. Supernatant (S) and eluted (E) fractions were resolved by 10% SDS-PAGE and visualized by Coomassie Blue staining.

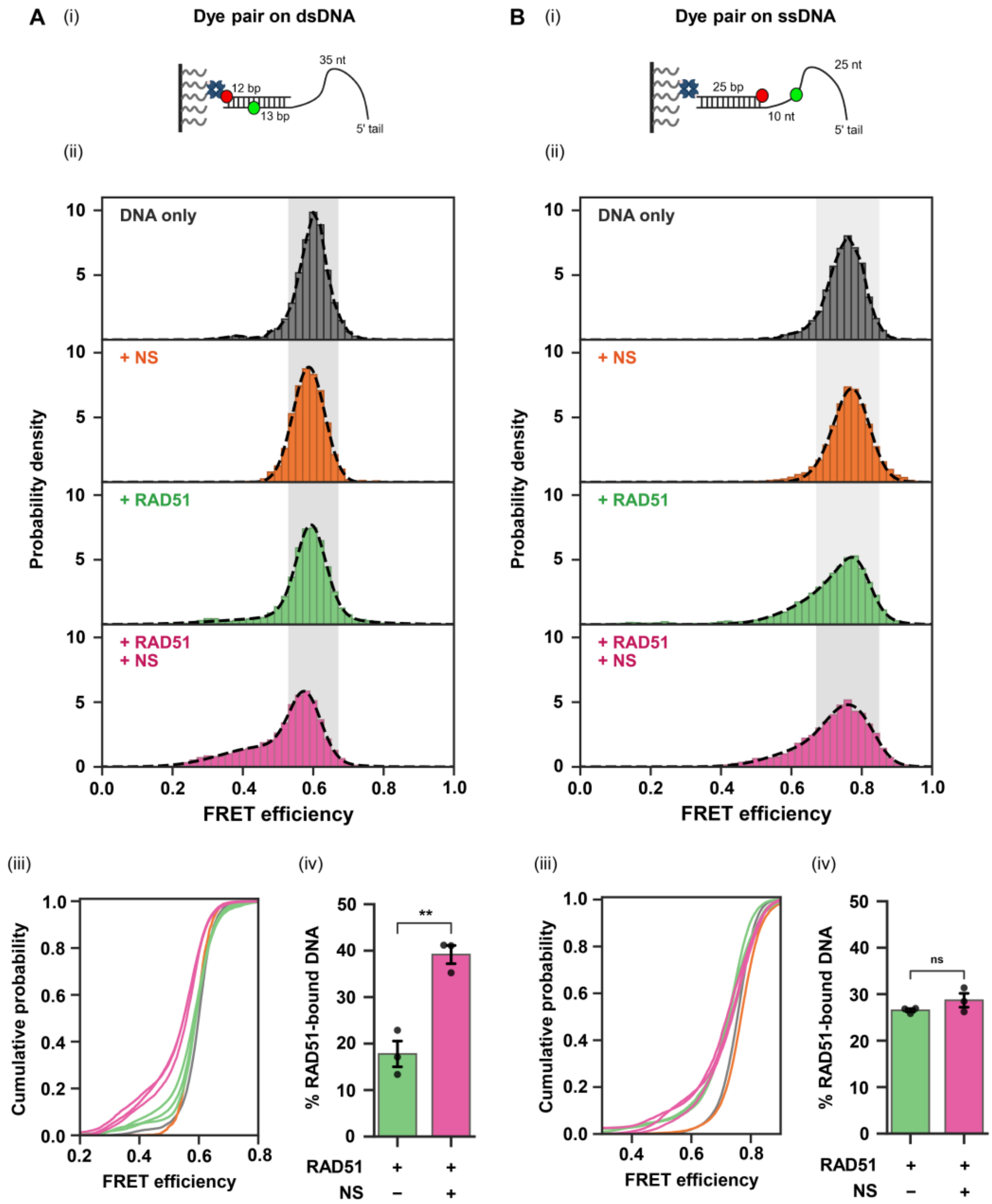

Supplementary Figure S7. NS recruits RAD51 to dsDNA. smFRET assay of RAD51 assembly on two DNA substrates with dye pairs located at **(A)** the distal double-stranded segment from the junction or **(B)** the ssDNA segment near the ssDNA/dsDNA junction. (i) A schematic of the experimental setup and DNA substrate. (ii) FRET histograms of DNA only, with NS alone, RAD51 alone, or the RAD51-NS mixture. The gray-shaded area represents the DNA-only state and its FRET is ~0.60 ± 0.07 (Supplementary Fig. S7A, panel ii) or ~0.76 ± 0.088 (Supplementary Fig. S7B, panel ii). (iii) The cumulative FRET probabilities from three independent experiments were plotted as unbinned empirical cumulative densities, with the same color scheme as used in the histograms (panel ii). (iv) RAD51-bound fractions were derived from the probabilities lower than the left bound of the grey-shaded DNA-only area in (ii), and quantified from three independent experiments with >250 total trajectories for each

condition (mean ± SEM). Statistical significance was determined by Student's *t*-test; ns (not significant): $P = 0.2329$; $^{**}P = 0.0032$.

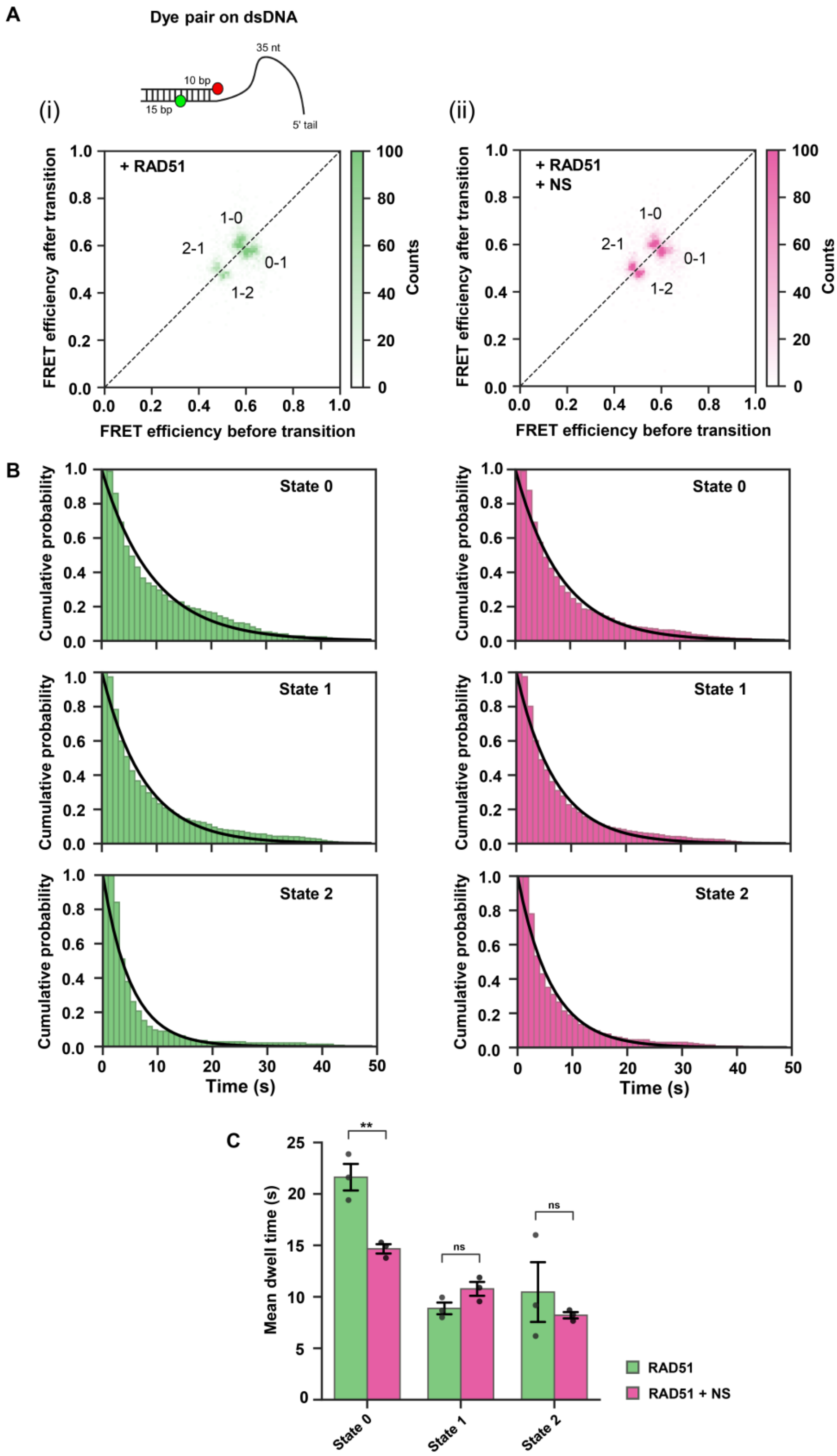

Supplementary Figure S8. Hidden Markov kinetic analysis of real-time smFRET time-courses on DNA substrate with dye pairs located at the double-stranded segment near the ssDNA/dsDNA junction. (**A**) Transition density plots of FRET efficiency before and after the transition for (i) RAD51 alone or (ii) the RAD51-NS mixture based

on the FRET time-courses. Symmetric peaks are shown along the diagonal dashed line, supporting the HMM assignment of FRET state. (**B**) Cumulative probability of dwell time for each FRET state. State 0: 0.7, State 1: 0.55, State 2: 0.4. Histograms were plotted by pooling all data from three independent experiments. The black curve represents the single-exponential estimate of dwell time. (**C**) Mean dwell time of each FRET state for RAD51 only (green bar) or the RAD51-NS mixture (red bar). Statistical significance was determined by Student's *t*-test; ns: not significant; State 0 group: ***P* = 0.0070; state 1 group: *P* = 0.0972; state 2 group: *P* = 0.4838.

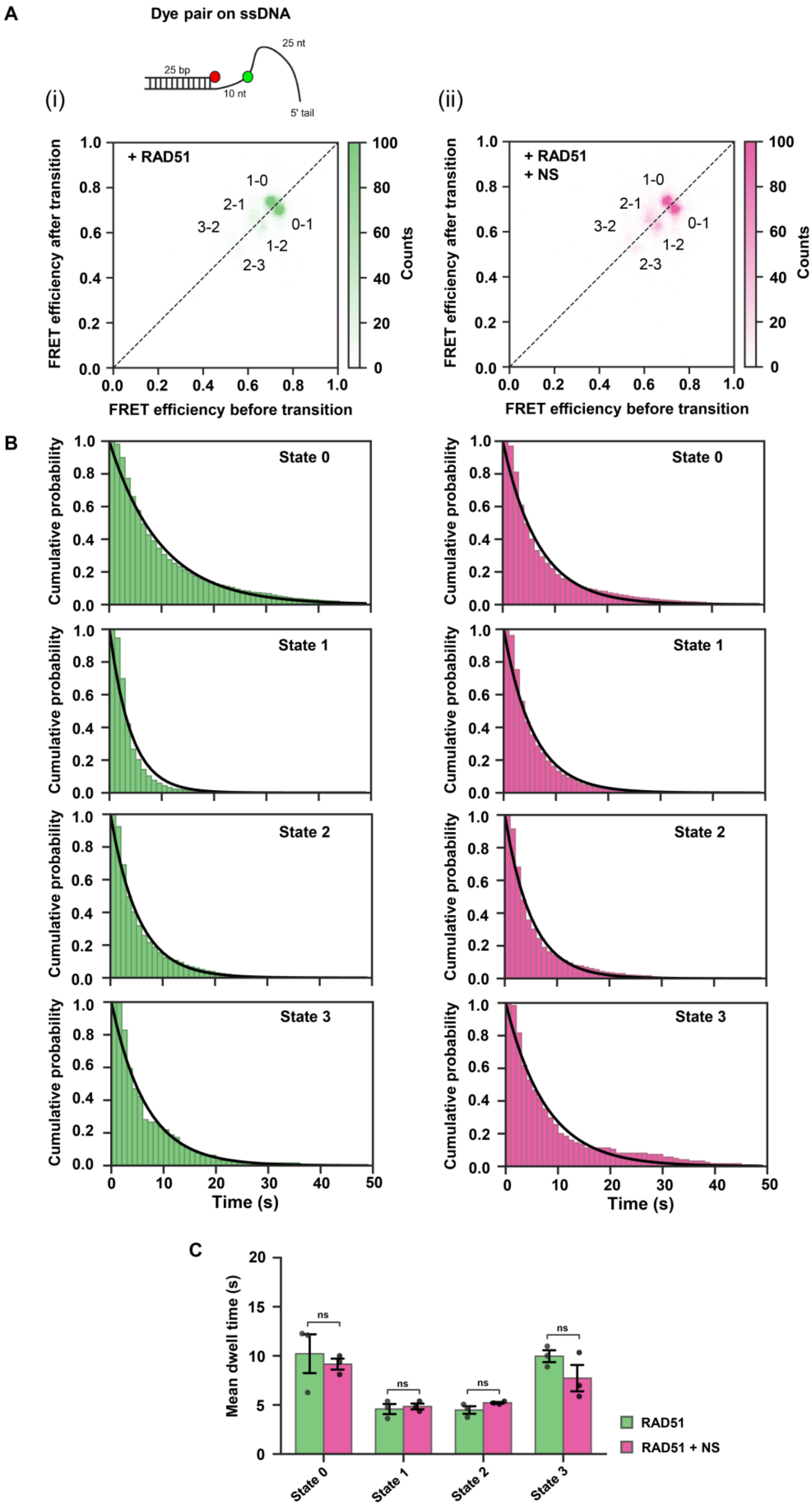

Supplementary Figure S9. Hidden Markov analysis of real-time smFRET time-

courses on DNA substrate with dye pairs located at the ssDNA segment near the ssDNA/dsDNA junction. (**A**) Transition density plots of FRET efficiency before and after the transition for (i) RAD51 alone or (ii) the RAD51-NS mixture based on the FRET time-courses. Symmetric peaks are shown along the diagonal dashed line, supporting the FRET state assignment. (**B**) Cumulative probability of dwell time for each FRET state. State 0: 0.76, State 1: 0.68, State 2: 0.59, State 3: 0.46. Histograms were plotted by pooling all data from three experiments. The black curve represents the single-exponential estimate of dwell time. (**C**) Mean dwell times of each FRET state for RAD51 only (green bar) or the RAD51-NS mixture (red bar). Statistical significance was determined by Student's *t*-test; ns: not significant; state 0 group: $P = 0.6338$; state 1 group: $P = 0.6777$; state 2: $P = 0.1379$; state 3: $P = 0.2036$.

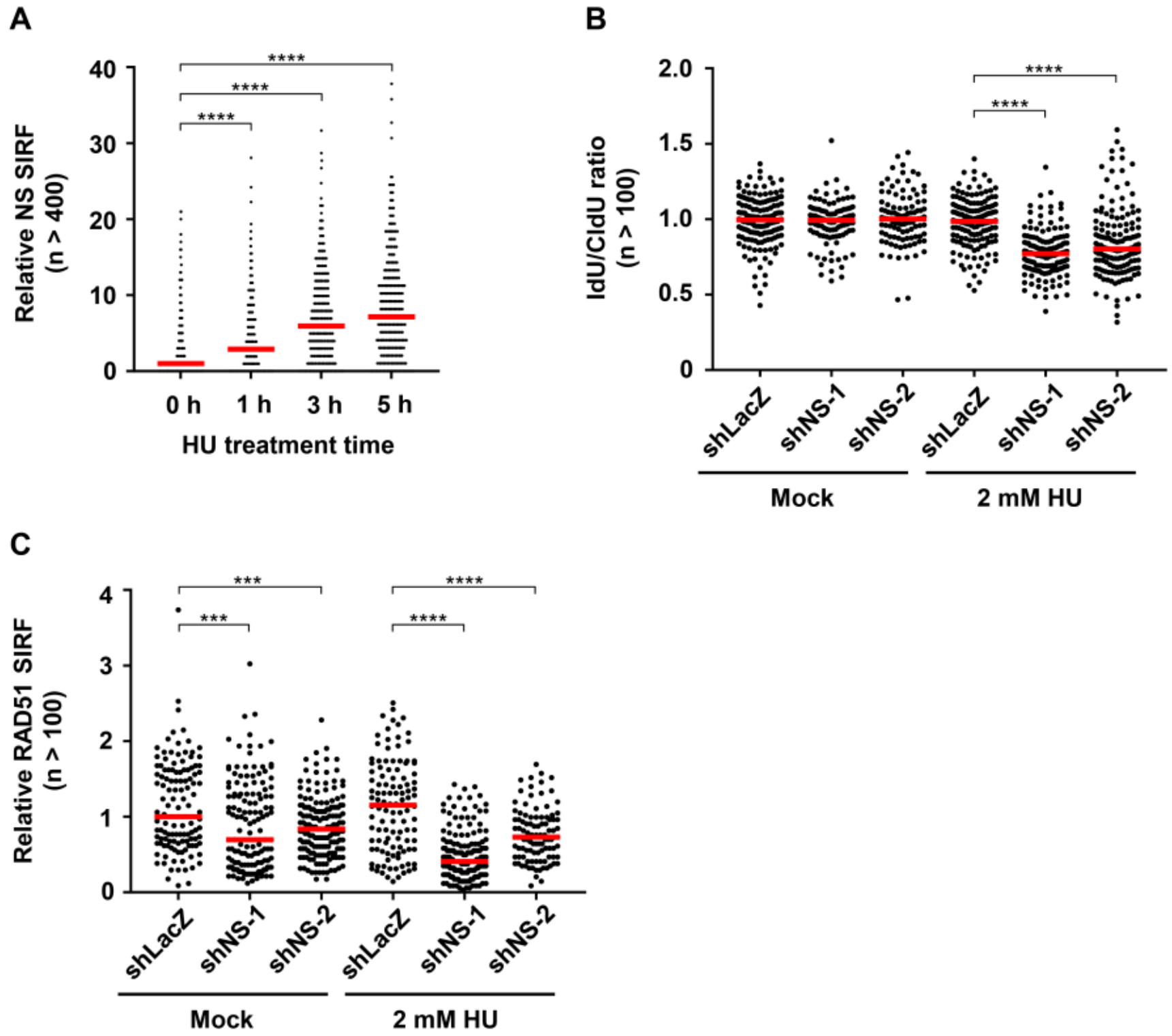

Supplementary Figure S10. Independent biological replicates of the cell-based experiments. **(A)** Single-cell SIRF-based quantification of NS at stalled forks as shown in Figure 1A. PLA foci per nucleus were quantified from >400 cells for each condition. **(B)** Fork stability during prolonged HU exposure as shown in Figure 1C. IdU:CldU ratios were quantified from >100 fibers. **(C)** Single-cell SIRF-based quantification of RAD51 at stalled forks as shown in Figure 4A. PLA foci per nucleus were quantified from >100 cells for each condition. All statistical significance was determined by the Mann-Whitney test. The *P* value under mock treatment between shLacZ and shNS-1 is ***$P = 0.0001$; between shLacZ and shNS-2 is ***$P = 0.0003$. Under HU treatment, all *P* values are ****$P < 0.0001$. The red line indicates the median.

Gene SYMBOL	Target sequence (5′ to 3′)	Clone ID
shLacZ	CGC GAT CGT AAT CAC CCG AGT	TRCN0000231722
shNS-1	GCT AAA CTG TTC TCT GTA TAA	TRCN0000074218
shNS-2	GTG CCT CAT TAG CTT ACT ATT	TRCN0000293681
shHLTF	GCA GGT GGA GTT GGT TTG AAT	TRCN0000272618
shBRCA1	GAG TAT GCA AAC AGC TAT AAT	TRCN0000244984
shSMARCAL1-1	AGG TGT TGA TTG GGT ACA ATG	TRCN0000236116
shSMARCAL1-2	CCA CAG TCC ACG TAG TCA AAT	TRCN0000236114

Supplementary Table S1. List of shRNA sequences.

Antibodies	WB dilution concentration	PLA dilution concentration	Catalog number	Source
Rabbit polyclonal anti-HLTF	1:1000	–	A300-230A	Bethyl
Mouse monoclonal anti-alpha-tubulin	1:5000	–	GTX628802-01	GeneTex
Rabbit polyclonal anti-BRCA1	1:5000	–	GTX100720	GeneTex
Mouse monoclonal anti-actin	1:5000	–	MAB1501	Millipore
Rabbit polyclonal anti-Nucleostemin	1:5000	1:200	ab70346	Abcam
Rabbit polyclonal anti-SMARCAL1	1:5000	–	A301-616A	Bethyl
Rabbit polyclonal anti-RAD51	–	1:100	ab63801	Abcam
Mouse monoclonal anti-PCNA	–	1:100	CBL407	Millipore
Mouse monoclonal anti-biotin	–	1:2000	200-002-211	Jackson ImmunoResearch
Rabbit polyclonal anti-biotin	–	1:3000	A150-109A	Bethyl
Mouse monoclonal anti-EB1	–	1:1000	610534	BD Bioscience
Mouse monoclonal anti-Flag (M2)	1:1000	–	F1804	Millipore
HRP anti-rabbit secondary antibody	1:10000	–	111-035-144	Jackson ImmunoResearch
HRP anti-mouse secondary antibody	1:10000	–	115-035-146	Jackson ImmunoResearch

Supplementary Table S2. List of antibodies used in this study.

Name	DNA sequence (5′ to 3′)
Oligo 1 (60 nt)	ACGCTGCCGAATTCTACCAGTGCCTTGCTAGGACATCTTT GCCCACCTGCAGGTTCACCC
Oligo 2 (60 nt)	Cy3-GGGTGAACCTGCAGGTGGGCAAAGATGTCCTAGCAA GGCACTGGTAGAATTCGGCAGCGT
Oligo 3 (60 nt)	A*C*G*C*T*GCCGAATTCTACCAGTGCCTTGCTAGGACAT CTTTGCCCACCTGCAGGTT*C*A*C*C*C
Oligo 4 (25 nt)	Cy3-GGGTGAACCTGCAGGTGGGCAAAGA
Oligo 5 (25 nt)	AGGCACTGGTAGAATTCGGCAGCGT-Cy3
Oligo 6 (55 nt)	Cy3-TTAGAGCTTAATTGCTGAATCTGGTGCTGT GGGTGAACCTGCAGGTGGGCAAAGA
Oligo 7 (90 nt)	A*C*G*C*T*GCCGAATTCTACCAGTGCCTTGCTAGGACAT CTTTGCCCACCTGCAGGTTCACCCTCTGTTTATTCATTTAC TTATTTTG*T*A*T*T*A
Oligo 8 (60 nt)	T*T*T*A*G*CTGCATATTTACAACATAGCGCAATACAGC ACCAGATTCAGCAATTAAGC*T*C*T*A*A
Oligo 9 (60 nt)	T*A*A*T*A*CAAAATAAGTAAATGAATAAACAGAATTGCG CTATGTTGTAAATATGCAG*C*T*A*A*A
Oligo 10 (25 nt)	Biotin-GGGTGAACCTGCAGGTGGGCAAAGA-Spacer9-Cy5
Oligo 11 (60 nt)	ACGCTGCCGAATTCTACCAGTGCCT-Cy3- TGCTAGGACATCTTTGCCCACCTGCAGGTTCACCC
Oligo 12 (59 nt)	ACGCTGCCGAATTCTACCAGTGCCTTGCTAGGACATCTT TGCCC-[Uni-Link™ Amino Modifier]-CCTGCAGGTTCACCC
Oligo 13 (25 nt)	Biotin-Cy5-Spacer9-GGGTGAACCTGCAGGTGGGCAAAGA
Oligo 14 (59 nt)	ACGCTGCCGAATTCTACCAGTGCCTTGCTAGGACATCTT TGCCCACC-[Uni-Link™ Amino Modifier]-GCAGGTTCACCC

Supplementary Table S3. Oligonucleotides utilized in this study.

Cy3: Cy3 fluorescence labeled on the 5′ or 3′ end of the indicated oligonucleotide.

* phosphorothioate bond modification between two nucleotides. nt: nucleotides.
